# Antibody Landscape Analysis following Influenza Vaccination and Natural Infection in Humans with a High-Throughput Multiplex Influenza Antibody Detection Assay

**DOI:** 10.1128/mBio.02808-20

**Published:** 2021-02-02

**Authors:** Zhu-Nan Li, Feng Liu, F. Liaini Gross, Lindsay Kim, Jill Ferdinands, Paul Carney, Jessie Chang, James Stevens, Terrence Tumpey, Min Z. Levine

**Affiliations:** a Influenza Division, National Center for Immunization and Respiratory Diseases, Centers for Disease Control and Prevention, Atlanta, Georgia, USA; b Division of Viral Diseases, National Center for Immunization and Respiratory Diseases, Centers for Disease Control and Prevention, Atlanta, Georgia, USA; c U.S. Public Health Service, Rockville, Maryland, USA; The Peter Doherty Institute for Infection and Immunity

**Keywords:** influenza, hemagglutinin, antibody, landscape, MIADA, human, ferret

## Abstract

To better understand the antibody landscape changes following influenza virus natural infection and vaccination, we developed a high-throughput multiplex influenza antibody detection assay (MIADA) containing 42 recombinant hemagglutinins (rHAs) (ectodomain and/or globular head domain) from pre-2009 A(H1N1), A(H1N1)pdm09, A(H2N2), A(H3N2), A(H5N1), A(H7N7), A(H7N9), A(H7N2), A(H9N2), A(H13N9), and influenza B viruses. Panels of ferret antisera, 227 paired human sera from vaccinees (children and adults) in 5 influenza seasons (2010 to 2018), and 17 paired human sera collected from real-time reverse transcription-PCR (rRT-PCR)-confirmed influenza A(H1N1)pdm09, influenza A(H3N2), or influenza B virus-infected adults were analyzed by the MIADA. Ferret antisera demonstrated clear strain-specific antibody responses to exposed subtype HA. Adults (19 to 49 years old) had broader antibody landscapes than young children (<3 years old) and older children (9 to 17 years old) both at baseline and post-vaccination. Influenza vaccination and infection induced the strongest antibody responses specific to HA(s) of exposed strain/subtype viruses and closely related strains; they also induced cross-reactive antibodies to an unexposed influenza virus subtype(s), including novel viruses. Subsequent serum adsorption confirmed that the cross-reactive antibodies against novel subtype HAs were mainly induced by exposures to A(H1N1)/A(H3N2) influenza A viruses. In contrast, adults infected by influenza B viruses mounted antibody responses mostly specific to two influenza B virus lineage HAs. Median fluorescence intensities (MFIs) and seroconversion in MIADA had good correlations with the titers and seroconversion measured by hemagglutination inhibition and microneutralization assays. Our study demonstrated that antibody landscape analysis by the MIADA can be used for influenza vaccine evaluations and characterization of influenza virus infections.

## Abstract (precis)

Repeated influenza vaccination and natural infections generate complex immune profiles in humans that require antibody landscape analysis to assess immunity and evaluate vaccines. However, antibody landscape analyses are difficult to perform using traditional assays.

## INTRODUCTION

There are 18 influenza A virus hemagglutinin (HA) subtypes, which are further divided into two phylogenetic groups: group 1 and group 2 (1–3). Influenza A(H1N1), influenza A(H3N2), and influenza B viruses cocirculate in the human population and cause seasonal epidemics. Vaccination is the most effective public health measure to control seasonal and potential pandemic influenza and reduce influenza morbidity and mortality (4, 5). Annual seasonal influenza vaccination is recommended in the United States; however, influenza vaccines need to be updated every season due to antigenic drift of the viruses. In addition, with antigenic drift and shift, novel zoonotic influenza viruses can emerge and cause outbreaks at human-animal interfaces, posing the risk of a pandemic.

Repeated influenza vaccinations and natural infections by historic and contemporary influenza viruses generate complex immune profiles that can be unique to each individual. It is thought that the first exposure to influenza viruses in life leaves an immunological imprint that can have profound impacts on the individual’s subsequent antibody responses to contemporary viruses (6–9). Understanding the complexity of the existing antibody immunity in the population by assessing the antibody landscape against both historic and contemporary viruses is crucial for the design of more effective vaccination strategies. However, such analysis requires the use of large numbers of viruses and antigens; traditional assays such as hemagglutination inhibition (HI) and virus microneutralization (MN) assays often have limited throughput and require the handling of live viruses under appropriate biological safety levels, which can restrict their applications (4, 10).

The HA globular head domain (GH HA1) contains more HA subtype-specific epitopes, whereas the HA stalk domain contains more shared epitopes that are cross-reactive within HA subtypes and/or groups (3, 11). Inactivated influenza vaccines (IIVs) mainly induce strain- or subtype-specific antibody responses that target the HA head domains. Recently, HA stalk-targeting universal vaccine candidates were developed and are under evaluation in clinical trials (12). Enzyme-linked immunosorbent assays (ELISAs) can be used to evaluate anti-influenza GH HA1 alone, whole HA (HA1 plus HA2), or neuraminidase (NA) binding antibodies (13). However, traditional ELISAs still can be time-consuming to perform and may require large volumes of sera when multiple antigens are tested.

We previously developed a Magpix assay for serologic diagnosis of seasonal and novel influenza virus infection (14, 15). In the current study, we expanded this platform and developed a high-throughput multiplex influenza antibody detection assay (MIADA) that can measure antibody responses to 42 HA antigens from 10 influenza virus subtypes simultaneously, plus a protein A (PA) control. Furthermore, we implemented an antibody adsorption technique using ectodomain (Ecto) HAs from A(H1N1) and A(H3N2) viruses to elucidate the effects of both homosubtypic (within-subtype) and heterosubtypic (cross-subtype) cross-reactivity (14, 15).

Here, using the 43-plex MIADA, we assessed antibody landscape changes in children and adults from the United States who received IIVs in 5 influenza seasons and in adults after influenza virus natural infections. We also demonstrated the correlation between the MIADA and HI/MN assays. Our results suggest that the MIADA will be a valuable tool for influenza vaccine evaluation and characterization of antibody responses to influenza virus infections.

## RESULTS

### MIADA can differentiate the antigenic differences between HAs of influenza viruses using ferret antisera.

To prepare the 43-plex MIADA, we selected representative recombinant hemagglutinins (rHAs) from current and previous seasonal epidemic subtypes: pre-2009 A(H1N1), A(H1N1)pdm09, A(H3N2), and influenza B viruses (B/Victoria lineage and B/Yamagata lineage). We also included an rHA from A(H2N2), the historic subtype that circulated in the human population from 1957 to 1968, and rHAs from several zoonotic influenza virus subtypes that caused recent animal outbreaks and human infections, including A(H5N1), A(H7N7), A(H7N9), A(H7N2), and A(H9N2). Finally, we included an rHA from A(H13N9), a subtype with no documented human infections, as a negative control (Table 1).

**TABLE 1:** Recombinant HAs of influenza viruses included in the 43-multiplex and antibody adsorptions

Virus strain	rHAs used in MIADA^*a*^								rHAs used in Ads^*b*^		
	Type (subtype/lineage)	Ecto/GH HA1	Abbreviation	HA group	Source	Egg or cell origin	GenBank accession no.	GISAID accession no.	Mock	2-Ads	8-Ads
A/South Carolina/1/1918	A(H1N1)	Ecto	H1.SC.18 E	1	CDC	Lung biopsy	AF117241	EPI5571			
A/Puerto Rico/8/1934	A(H1N1)	Ecto	H1.PR.34 E	1	CDC	Unknown	AF389118	EPI252235			
A/Marton/1943	A(H1N1)	Ecto	H1.Mar.43 E	1	CDC	Egg	CY020285	EPI240837			
A/USSR/90/1977	A(H1N1)	Ecto	H1.USS.77 E	1	CDC	Egg	CY121878	EPI390455	N		Y
A/Taiwan/01/1986	A(H1N1)	Ecto	H1.Tw.86 E	1	CDC	Cell	JF816653	EPI318034	N		Y
A/Texas/36/1991	A(H1N1)	Ecto	H1.Tx.91 E	1	CDC	Egg	CY033655	EPI159432			
A/New Caledonia/20/1999	A(H1N1)	Ecto	H1.NC.99 E	1	CDC	Unknown	AY289929	EPI18473	N		Y
A/Brisbane/59/2007	A(H1N1)	Ecto	H1.BR.07 E	1	CDC	Cell	CY030230	EPI162498			
A/California/07/2009	A(H1N1)	Ecto	H1.CA.09 E	1	IRR	Cell	FJ981613	EPI177294	N	Y	Y
A/California/07/2009	A(H1N1)	GH HA1	H1.CA.09 G	1	CDC	Cell	FJ981613	EPI177294			
A/Michigan/45/2015	A(H1N1)	Ecto	H1.MI.15 E	1	CDC	Egg	KU933493	EPI685579			
A/Japan/305/1957	A(H2N2)	GH HA1	H2.Jap.57 G	1	IRR	Unknown	AAO46269	EPI128485			
A/Hong Kong/8/1968	A(H3N2)	Ecto	H3.HK.68 E	2	CDC	Unknown	CY044261	EPI240947			
A/Port Chalmers/1/1973	A(H3N2)	Ecto	H3.PC.73 E	2	CDC	Egg	CY113109	EPI365202			
A/Victoria/3/1975	A(H3N2)	Ecto	H3.VC.75 E	2	CDC	Unknown	V01086	EPI131278			
A/Bangkok/1/1979	A(H3N2)	Ecto	H3.BK.79 E	2	CDC	Egg	CY121000	EPI377537	N		Y
A/Leningrad/360/1986	A(H3N2)	Ecto	H3.LN.86 E	2	CDC	Egg	CY121277	EPI385984			
A/Shanghai/11/1987	A(H3N2)	Ecto	H3.SH.87 E	2	CDC	Egg	CY121309	EPI390002			
A/Beijing/32/1992	A(H3N2)	Ecto	H3.BJ.92 E	2	CDC	Cell	CY113677	EPI365898	N		Y
A/Johannesburg/33/1994	A(H3N2)	Ecto	H3.JH.94 E	2	CDC	Egg	CY121341	EPI390018			
A/Nanchang/933/1995	A(H3N2)	Ecto	H3.NC.95 E	2	CDC	Cell	CY112789	EPI362794			
A/Sydney/5/1997	A(H3N2)	Ecto	H3.Syd.97 E	2	CDC	Cell	CY112885	EPI362863			
A/Panama/2007/1999	A(H3N2)	Ecto	H3.Pan.99 E	2	CDC	Unknown	DQ508865	EPI105036			
A/Wyoming/03/2003	A(H3N2)	Ecto	H3.WY.03 E	2	CDC	Cell	EU268227	EPI152388			
A/California/7/2004	A(H3N2)	Ecto	H3.CA.04 E	2	CDC	Cell	CY114373	EPI367105			
A/Wisconsin/67/2005	A(H3N2)	GH HA1	H3.WI.05 G	2	CDC	Cell	DQ865947	EPI106424			
A/Perth/16/2009	A(H3N2)	Ecto	H3.Per.09 E	2	IRR	Cell	ACS71642	EPI182941	N	Y	Y
A/Perth/16/2009	A(H3N2)	GH HA1	H3.Per.09 G	2	IRR	Cell	ACS71642	EPI182941			
A/Victoria/361/2011	A(H3N2)	Ecto	H3.VC.11 E	2	CDC	Cell		EPI349103			
A/Texas/50/2012	A(H3N2)	Ecto	H3.Tx.12 E	2	CDC	Cell		EPI398417			
A/Switzerland/9715293/2013	A(H3N2)	Ecto	H3.SW.13 E	2	CDC	Cell		EPI530687			
A/Maryland/26/2014	A(H3N2)	Ecto	H3.MD.14 E	2	CDC	Cell		EPI550983	N		Y
A/Vietnam/1203/2004	A(H5N1)	GH HA1	H5.VN.04 G	1	IRR	Unknown	HM006759	EPI361524			
A/Indonesia/5/2005	A(H5N1)	Ecto	H5.Ind.05 E	1	IRR	Egg	CY116646	EPI376537			
A/Indonesia/05/2005	A(H5N1)	GH HA1	H5.Ind.05 G	1	CDC	Egg	CY116646	EPI376537			
A/Netherlands/219/2003	A(H7N7)	GH HA1	H7.NED.03 G	2	IRR	Unknown	AAR02640	EPI18992			
A/Shanghai/2/2013	A(H7N9)	GH HA1	H7.SH.13 G	2	CDC	Egg		EPI439502			
A/New York/108/2016	A(H7N2)	Ecto	H7.NY.16 E	2	CDC	Egg	KY888124	EPI944629			
A/Hong Kong/33982/2009	A(H9N2)	GH HA1	H9.HK.09 G	1	IRR	Cell	KF188316	EPI470900			
A/shorebird/DE/68/2004	A(H13N9)	GH HA1	H13.DE.04 G	1	IRR	Egg	CY005931	EPI744939			
B/Brisbane/60/2008	B(Victoria)	GH HA1	B.B.08 G	NA	IRR	Egg	FJ766840	EPI173277			
B/Wisconsin/01/2010	B(Yamagata)	GH HA1	B.W.10 G	NA	IRR	Cell	JN993010	EPI363743			
Protein A	NA	NA	PA	NA	Fisher Scientific	NA	NA	NA			

a Recombinant ectodomain and/or globular head (GH) HA1 rHAs were used in this study. NA, not applicable; CDC, Centers for Disease Control and Prevention; IRR, International Reagent Resource.

b Y (yes) and N (no) represent rHAs that were included and not included, respectively, in 2-antigen or 8-antigen serum adsorptions.

All samples and control serum pools were tested in duplicates. The MIADA assay can generate antibody landscapes with very small volumes of sera (10 μl per sample) with <10% coefficient of variation (CV) for intra-assay and <15% CV for inter-assay variations (data not shown). In order to determine the optimal dilution of sera, we assessed the linearity of the assay using paired vaccination sera collected from the 2018–2019 influenza season. The MIADA has a wide linear range that spanned from 10 to 10^3^ or 10^4^ serum dilutions for most rHAs (see Fig. S1 and S2 in the supplemental material) (16). In our previous studies, we used a serum dilution of 1:40 to detect influenza virus antibody responses using the same assay platform (14, 15, 17). Here, the linearity analysis suggests that the 1:40 serum dilution falls well within the linear range for most seasonal and novel subtype influenza virus antigens and is suitable to capture the full scope of the antibody responses to most antigens while maintaining the assay throughput. Thus, this serum dilution was used for the antibody landscape analysis. We then compared signals obtained from the 43-plex versus the 1-plex assay using 9 pairs of vaccination sera collected in 2018 to 2019. There was no significant reduction in the mean fluorescence intensity (MFI) signals detected in the 43-plex versus those in the 1-plex assay. The differences in MFI values detected between the 43-plex and 1-plex assays were less than 20% for most antigens (Fig. S3).

10.1128/mBio.02808-20.1 FIG S1 Linearity of the MIADA. A total of 9 paired sera from vaccine recipients in 2018 to 2019 were tested in duplicates at serial 2-fold dilutions starting from 1:10 to 1:81,920 by the multiplex MIADA. (A) Pre-vaccination (S1) sera; (B) post-vaccination (S2) sera. Responses to virus HA antigens are color coded by subtypes: H1, H2, H3, H7, H5, H9, H13, influenza B virus, and protein A. Download FIG S1, PDF file, 0.4 MB. Copyright © 2021 Li et al. 2021 Li et al. This content is distributed under the terms of the Creative Commons Attribution 4.0 International license.

10.1128/mBio.02808-20.2 FIG S2 Linear range of MFIs in MIADA pre- and post-vaccination. A total of 9 paired sera from vaccine recipients in 2018 to 2019 were tested in duplicates at serial 2-fold dilutions starting from 1:10 to 1:81,920 by the MIADA. MFIs pre-vaccination (S1) and post-vaccination (S2) to 8 antigens were plotted together for each vaccine recipient to demonstrate the linear range of the responses. PA, protein A. Download FIG S2, PDF file, 0.3 MB. Copyright © 2021 Li et al. 2021 Li et al. This content is distributed under the terms of the Creative Commons Attribution 4.0 International license.

10.1128/mBio.02808-20.3 FIG S3 Comparison of MFI values between the 43-plex and 1-plex MIADA. A total of 9 paired sera from vaccine recipients in 2018 to 2019 were tested in duplicates at a 1:40 dilution by the MIADA assay. The means and standard deviations of MFIs to seven representative antigens from two independent experiments are plotted. (A) S1 (pre-vaccination); (B) S2 (post-vaccination). Download FIG S3, PDF file, 0.09 MB. Copyright © 2021 Li et al. 2021 Li et al. This content is distributed under the terms of the Creative Commons Attribution 4.0 International license.

Ferret antisera are used by the World Health Organization (WHO) influenza collaborating centers in either HI or MN assays to characterize the antigenic drift of influenza viruses for global influenza surveillance. To evaluate whether ferret antisera (Table S1) can also detect antigenic differences between the rHAs used in the MIADA, we performed a large antigenic characterization using all 42 rHAs in the current study. As shown in Table 2, all ferret antisera were able to detect the highest MFIs against rHAs from their homologous viruses or the most closely related virus strains, with reduced MFIs to rHAs from viruses that are antigenically drifted but within the same subtype; the highest reductions of MFIs were for rHAs from different subtypes. These results suggested that the MIADA could detect antigenic differences between these antigens (Table 2). Of note, some hetero-subtypic cross-reactivity was also detected; for example, ferret anti-H2N2 (A/Japan/305/1957 [Japan/1957]) serum cross-reacted with A(H1N1) HAs within group 1 (Table 2).

**TABLE 2:**
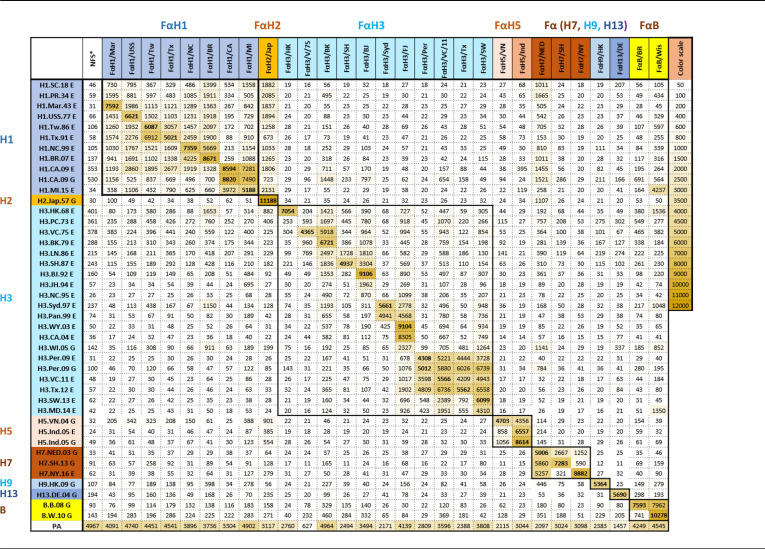
Antigenic characterization of HA strain/subtype-specific antibody responses using ferret antisera in MIADA^*a*^

a *, normal ferret sera.

10.1128/mBio.02808-20.4 TABLE S1 Ferret antisera used in this study. Details of the ferret sera used for antigenic characterization (Table 2) are listed. Download Table S1, PDF file, 0.1 MB. Copyright © 2021 Li et al. 2021 Li et al. This content is distributed under the terms of the Creative Commons Attribution 4.0 International license.

### Antibody landscapes shifted following influenza vaccination in humans.

**FIG 1:**
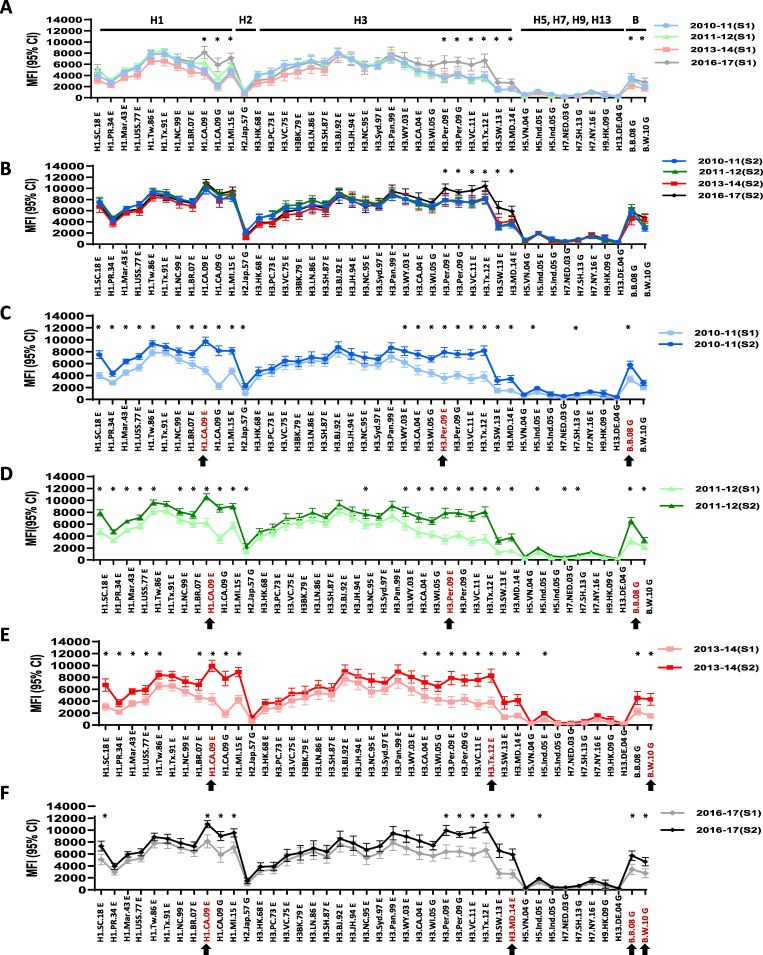
Antibody landscapes in adults shifted following inactivated influenza vaccination (IIV). S1 and S2 serum samples collected from adult IIV recipients from 4 seasons from 2010 to 2016 were tested by the MIADA. (A) S1 only from 4 seasons (*n* = 162); (B) S2 only from 4 seasons (*n* = 162); (C) 2010 to 2011 (S1 versus S2) (*n* = 49); (D) 2011 to 2012 (S1 versus S2) (*n* = 58); (E) 2013 to 2014 (S1 versus S2) (*n* = 27); (F) 2016 to 2017 (S1 versus S2) (*n* = 28). Arrows indicate rHAs of the vaccine viruses or most closely related to the vaccine viruses. The *y* axis shows mean MFIs with 95% confidence intervals (CI). Antibody responses measured by MFIs to HAs from each strain were compared between seasons (A and B) or within the same season (C to F). *, *P < *0.05.

To assess the antibody landscape changes following influenza vaccination, we first analyzed pre-vaccination (S1) and post-vaccination (S2) sera collected from adults (19 to 49 years of age) who received inactivated influenza vaccination in 4 influenza seasons: 2010 to 2011, 2011 to 2012, 2013 to 2014, and 2016 to 2017 (Table 3 and Fig. 1). In the MIADA, we incorporated rHAs from 11 representative historic A(H1N1) viruses isolated from 1918 (A/South Carolina/1/1918) to the vaccine virus in the 2018–2019 season (A/Michigan/45/2015) and from 20 representative A(H3N2) viruses from 1968 (A/Hong Kong/8/68) to A/Maryland/26/2014 (a Hong Kong/4801/2014-like virus, similar to the 2016–2017 vaccine component) (Table 1). Antibody landscape changes from pre- and post-vaccination to 42 rHA antigens were measured simultaneously by the MIADA (Fig. 1). In general, vaccination induced the strongest antibody responses to the vaccine antigens (A/H1N1, A/H3N2, and B viruses); it also boosted cross-reactive antibodies to antigens from viruses in the adjacent antigenic clusters (Fig. 1). In adults, when overlaying the pre-vaccination (S1) baseline antibodies from 4 influenza seasons from 2010 to 2011 through 2016 to 2017, baseline antibodies to rHAs from the recent epidemic A(H1N1), A(H3N2), and B virus strains increased in more recent seasons (for example, 2016 to 2017 versus 2010 to 2012 [*P < *0.05]) but not for novel influenza viruses that were not circulating in the United States (*P > *0.05) (Fig. 1A). Following vaccination, the shapes of the antibody landscapes among adults from each of the 4 seasons were surprisingly similar (*n* = 49 for 2010 to 2011, *n* = 58 for 2011 to 2012, *n* = 27 for 2013 to 2014, and *n* = 28 for 2016 to 2017) (Fig. 1B to F and Table S2), except that more elevated MFIs to rHAs from contemporary A(H3N2) viruses were detected in the 2016–2017 season (Fig. 1B) (*P < *0.05), likely due to the higher baseline S1 values (Fig. 1A and F and Table S2). The analysis of the delta values (S2 − S1) suggested that the highest antibody rises were to the rHAs from the contemporary viruses closely related to the vaccine strains (Table S2).

**TABLE 3:** Characteristics of the vaccinees in the study and the components of inactivated influenza vaccines

IIV season	No. of paired samples	No. of samples from vaccinees at age^*a*^ of:			Influenza virus strains			
		0–3 yrs	9–17 yrs	19–49 yrs	H1N1	H3N2	B (Victoria lineage)	B (Yamagata lineage)
2010–2011	49	0	0	49	A/California/07/2009	A/Perth/16/2009	B/Brisbane/60/2008	NA
2011–2012	58	0	0	58	A/California/07/2009	A/Perth/16/2009	B/Brisbane/60/2008	NA
2013–2014	27	0	0	27	A/California/07/2009	A/Texas/50/2012	NA	B/Massachusetts/2/2012
2016–2017	28	0	0	28	A/California/07/2009	A/Hong Kong/4801/2014	B/Brisbane/2008	B/Phuket/3073/2013
2018–2019	65	22	22	21	A/Michigan/45/2015	A/Singapore/INFIMH-16-0019/2016	B/Colorado/06/2017	B/Phuket/3073/2013

Total	227	22	22	183				

a Age at the time of serum collection.

10.1128/mBio.02808-20.5 TABLE S2 MFI delta values between pre-vaccination (S1) and post-vaccination (S2) in 2010–2018 and between preinfection (S1) and postinfection (S2) in 2016–2018 seasons. Delta values were calculated as S2 − S1. The color scale indicates MFI levels. Download Table S2, PDF file, 0.1 MB. Copyright © 2021 Li et al. 2021 Li et al. This content is distributed under the terms of the Creative Commons Attribution 4.0 International license.

**FIG 2:**
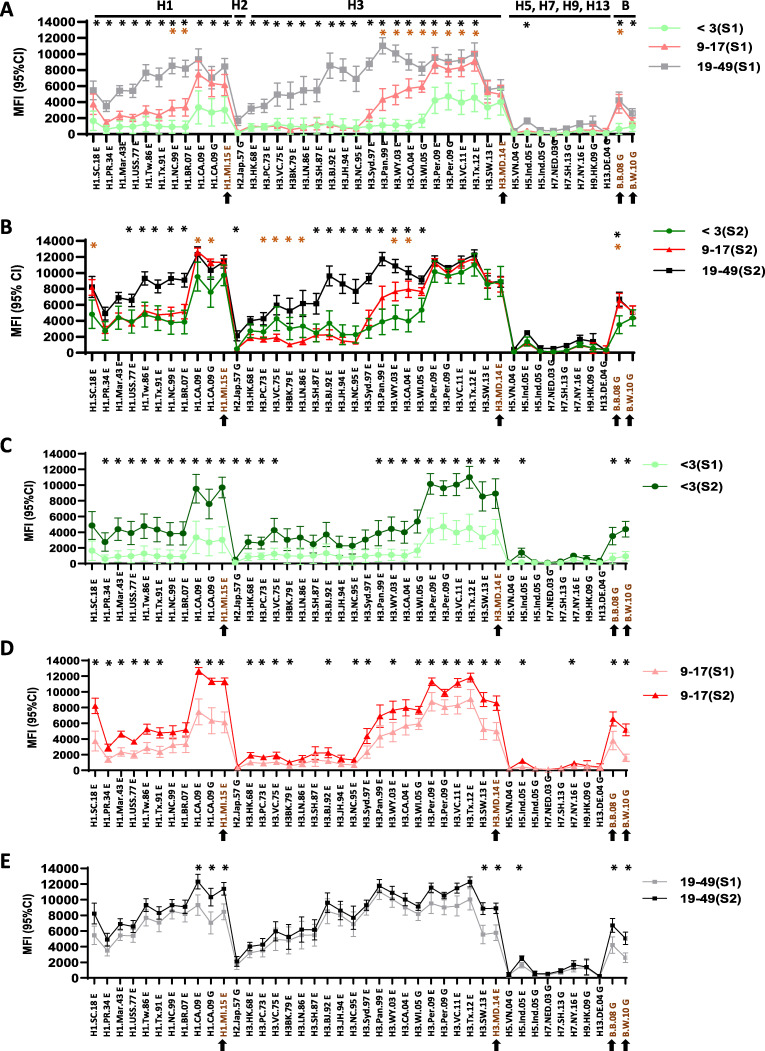
Age-related differences in antibody landscapes in young children, older children, and adults following IIV. Pre-vaccination (S1) and post-vaccination (S2) serum samples were collected from 2018–2019 IIV recipients in 3 age groups. (A) S1 among 3 age groups; (B) S2 among 3 age groups; (C) S1 and S2 in young children (<3 years old) (*n* = 22); (D) S1 and S2 in older children (9 to 17 years old) (*n* = 22); (E) S1 and S2 in adults (19 to 49 years old) (*n* = 21). Arrows indicate rHAs of the vaccine viruses or most closely related to the vaccine viruses. The *y* axis shows mean MFI values with 95% confidence intervals (CI). MFIs in S1 and S2 was compared between age groups in panels A and B. Black asterisks indicate a *P* value of *<*0.05 comparing <3-year-old versus 19- to 49-year-old vaccinees; red asterisks indicate a *P* value of *<*0.05 comparing <3-year-old versus 9- to 17-year-old vaccinees. Vaccine response (S1 versus S2) for each age group are compared in panels C to E. Black asterisks indicate a *P* value of *<*0.05.

To understand how age can impact the antibody responses to vaccination, we then analyzed the antibody landscapes in the 2018–2019 influenza season among three age groups who received quadrivalent inactivated influenza vaccination (Table 3 and Fig. 2): very young children (<3 years old), older children (9 to 17 years old), and adults (19 to 49 years old). There was a stark difference in the antibody landscapes for pre-2009 A(H1N1), A(H1N1)pdm09, A(H3N2), and influenza B viruses between the age groups (Fig. 2). At baseline, young children (<3 years old) exhibited a narrower antibody landscape than did older children (9 to 17 years old) and adults (*P < *0.05), with the majority of their antibody responses targeting rHAs from contemporary viruses (Fig. 2A). In contrast, adults had higher levels of pre-existing antibodies to rHAs from pre-2009 A(H1N1), A(H1N1)pdm09, and A(H3N2) viruses (*P < *0.05), displaying a much broader antibody landscape (Fig. 2A). This reflected the increasing complexity of influenza exposures with age. This age-related difference in the antibody landscapes was largely retained following vaccination (Fig. 2), although post-vaccination antibodies to rHAs from the vaccine virus and recent circulating viruses reached similar levels in all three age groups (Fig. 2B). Of note, young children (<3 years old) showed slightly higher MFIs against some of the early H3 rHAs from 1970 to the 1980s than did the older pediatric group (9 to 17 years old) (Fig. 2B) (*P < *0.05). This could be due to the higher antibody rise post-vaccination in young children (<3 years old) that may have induced broader cross-reactivity, as evidenced by their high A(H3N2) MFI delta values compared to those from the older children (9 to 17 years old) (S2 − S1) (Table S2). As expected, there were very little antibody responses to HAs from the novel viruses (H5, H7, H9, and H13 mean MFIs of <1,300) to which the participants had no exposures (Fig. 1, Fig. 2C to E, and Table S2).

### Antibody landscapes shifted following influenza virus natural infection in humans.

**FIG 3:**
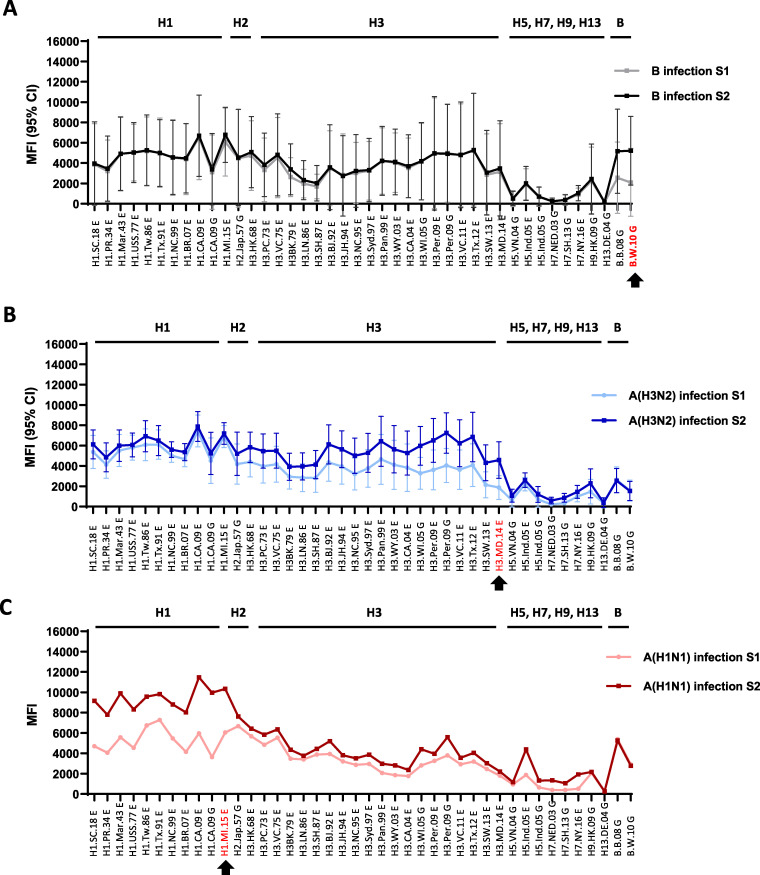
Antibody landscape shifted following influenza virus natural infections. Acute (S1)- and convalescent (S2)- phase sera collected from 17 rRT-PCR-confirmed influenza A or B virus-infected persons were tested by MIADA. (A) Pre- and post-infection landscapes of influenza B virus infections (*n* = 5); (B) pre- and post-infection landscapes of A(H3N2) infections (*n* = 10); (C) pre- and post-infection landscapes of A(H1N1)pdm09 infections (*n* = 2). Arrows indicate HA of the infecting virus strain or the most closely related HA to the infecting strain. The *y* axis shows mean MFI values, and 95% confidence intervals (CI) are indicated in panels A and B (*n* > 2).

Next, we analyzed antibody landscapes (Fig. 3) and delta values of MFIs (Table S2) from acute (S1)- and convalescent (S2)-phase sera collected from 17 adults with real-time reverse transcription-PCR (rRT-PCR)-confirmed influenza virus infections in 2016–2018 seasons: 5 from influenza B, 10 from A(H3N2), and 2 from A(H1N1)pdm09 virus infections (Table 4). In influenza B virus-infected persons, only MFIs against GH HA1 rHAs from 2 influenza B lineage viruses increased in the convalescent-phase sera compared to those in the acute-phase sera (Fig. 3A and Table S2), with little to no cross-reactivity to rHAs from influenza A viruses. Surprisingly, in influenza A virus-infected cases, although antibodies in the convalescent-phase sera showed the highest increase to the rHAs from the infecting or closely related strains, antibodies to other influenza A virus strains also increased to various degrees (Fig. 3B and C and Table S2). In A(H3N2) virus-infected persons, infection with A/Hong Kong/4801/2014-like viruses induced antibody responses to rHAs from all A(H3N2) strains between 1968 and 2014; slight antibody rises to other novel subtype HAs were also observed (Fig. 3B and Table S2). Furthermore, the two persons infected with A/Michigan/15/2015-like A(H1N1)pdm09 viruses not only had increased antibodies to rHAs from all A(H1N1) strains in their convalescent-phase sera, but also showed elevated titers against rHAs from some A(H3N2) viruses and novel subtype viruses, including H5 and H7 (Fig. 3C and Table S2). A(H1N1)pdm09 and A(H3N2) virus-infected cases did not induce any cross-reactive antibodies to rHAs from influenza B viruses (Table S2).

**TABLE 4:** Detection of influenza virus natural infection determined by seroconversion in MIADA versus HI/MN assays

Infecting virus strain	No. of cases^*a*^	Median age (yrs) (range)^*b*^	Median no. of days between S1 and S2 (range)	Median no. of days of S1 collection from symptom onset (range)	% seroconversion by HI/MN (no. of cases that seroconverted/total no. of cases)^*c*^	% seroconversion by MIADA (no. of cases that seroconverted/total no. of cases)^*d*^
B	5	70 (55–75)	26 (21–32)	5 (1–7)	40 (2/5)	40 (2/5)
A(H3N2)	10	72 (52–90)	25 (16–31)	6 (2–17)	70 (7/10)	60 (6/10)
A(H1N1)pdm09	2	67 (60–74)	27 (23–30)	5 (3–6)	50 (1/2)	50 (1/2)

a Influenza virus-infected persons confirmed by rRT-PCR in the 2016–2018 influenza seasons.

b Age at the time of serum collection.

c Seroconversion was determined by a ≥4-fold rise in HI titers [for influenza B and A(H1N1) viruses] or MN titers [for A(H3N2)] compared to positivity determined by rRT-PCR.

d Seroconversion was determined by a ≥2-fold rise in MFIs against HA of the same or the most closely related virus strain compared to positivity determined by rRT-PCR.

### A(H1N1)pdm09 and A(H3N2) natural infection induced cross-reactive antibodies to novel influenza virus subtypes that can be removed by serum adsorption.

From the antibody landscape analysis, we observed MFI rises to H5, H7, H9, and H13 novel subtype rHAs following infection with seasonal influenza viruses (Table S2). To understand whether antibodies to these novel subtype influenza viruses from infection cases were caused by cross-reactive responses to seasonal influenza viruses that the persons may have been exposed to in the past, we performed serum adsorption analysis. We used adsorption first with two ectodomain (Ecto) rHAs from A(H1N1)pdm09 strain A/California/07/2009 and A(H3N2) strain A/Perth/16/2009 (2-Ads) and then with eight ectodomain rHAs from four historic pre-2009 A(H1N1) and four historic A(H3N2) strains (8-Ads) (Table 1).

To determine seroconversion in the MIADA for paired serum samples, we used a ≥2-fold rise in MFIs after adjustment of low S1 MFIs to 1,000 (14, 15). We found that one A(H1N1)pdm09-infected person and three A(H3N2)-infected persons achieved seroconversion (≥2‐fold rises in MFIs) against at least one novel subtype rHA (H5, H7, H9, or H13) (Tables S3 and S4), and nine persons showed high MFIs (≥2,000) against a novel subtype rHA(s) in S2 and/or S1 sera, although no seroconversions in MFIs were achieved (Table S5 and data not shown).

10.1128/mBio.02808-20.6 TABLE S3 MFI values against novel subtype HAs after mock adsorption (Ad) or 2-Ads among A(H1N1)pdm09 and A(H3N2) infection cases with MFI seroconversion to novel subtype influenza viruses. The color scale indicates MFI levels. Download Table S3, PDF file, 0.09 MB. Copyright © 2021 Li et al. 2021 Li et al. This content is distributed under the terms of the Creative Commons Attribution 4.0 International license.

10.1128/mBio.02808-20.7 TABLE S4 Fold rise after mock adsorption or 2-Ads among A(H1N1)pdm09 and A(H3N2) infection cases with MFI seroconversion to novel subtype influenza viruses. The color scale indicates fold rise levels. Download Table S4, PDF file, 0.08 MB. Copyright © 2021 Li et al. 2021 Li et al. This content is distributed under the terms of the Creative Commons Attribution 4.0 International license.

10.1128/mBio.02808-20.8 TABLE S5 MFIs against novel subtype Ecto and/or GH HA1 detected in 9 S2 sera were partially removed by 2-Ads or 8-Ads. The color scale indicates MFI levels. Download Table S5, PDF file, 0.10 MB. Copyright © 2021 Li et al. 2021 Li et al. This content is distributed under the terms of the Creative Commons Attribution 4.0 International license.

First, four paired and nine single S2 sera were either mock adsorbed (adsorption with beads only [no rHAs]) or adsorbed with two ectodomain rHAs (2-Ads). Seroconversion in MFIs against novel subtype rHAs was eliminated in three out of four paired samples (Table S4), and two out of nine S2 sera (case F and case G) also showed MFIs of <1,000 against all novel subtype rHAs after 2-Ads (Table S5). Only one A(H1N1) virus infection case (case A) still showed a >2-fold rise against H5.Ind.05 E after 2-Ads (Table S4).

**FIG 4:**
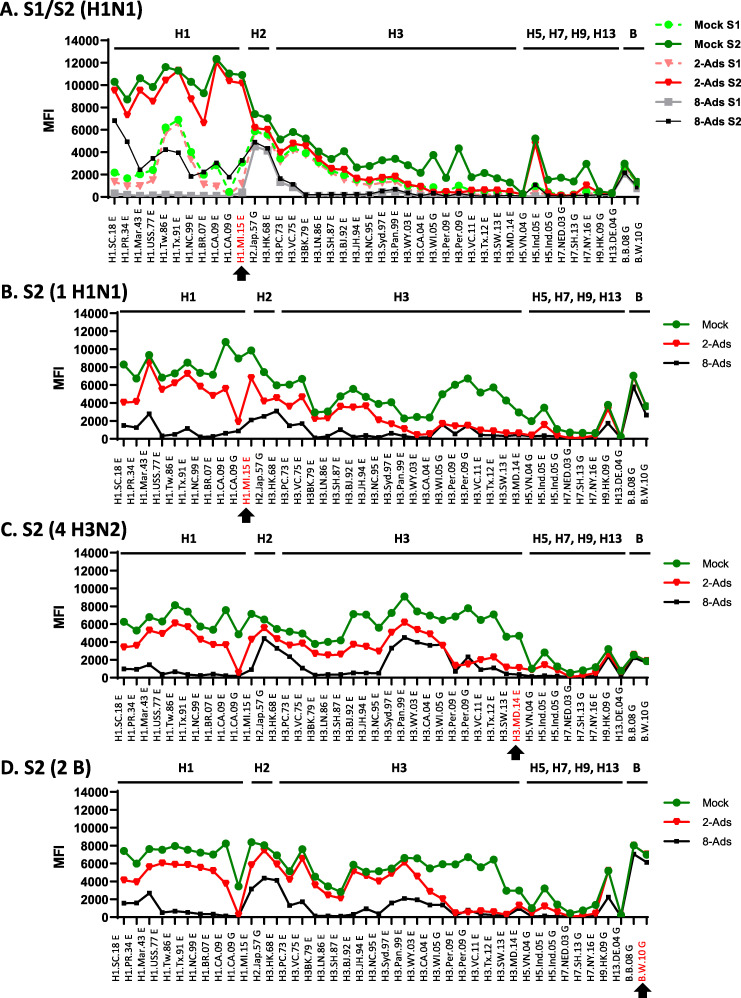
Cross-reactive antibodies against novel subtype HA(s) due to exposures to A(H1N1)pdm09 and/or A(H3N2) viruses were confirmed by serum adsorption. Antibody landscape analysis of influenza A and B virus infection sera following mock, 2-Ads, or 8-Ads was performed. (A) S1 (acute-phase) and S2 (convalescent-phase) sera from one A(H1N1)pdm09 virus-infected person that showed seroconversion to novel subtype HA; (B) one S2 serum sample from the A(H1N1)pdm09 case; (C) four S2 sera from A(H3N2) cases; (D) two S2 sera from influenza B virus cases.

Next, one set of paired sera from an A(H1N1)pdm09-infected person (case A) and seven S2 sera from one A(H1N1)pdm09 (case E), four A(H3N2) (cases H to K), and two influenza B (cases L to M) virus cases (MFI of ≥2,000 against novel subtype HAs after 2-Ads) were further adsorbed by eight ectodomain rHAs (8-Ads) (Table 1, Fig. 4, and Table S5). Antibody landscapes shifted significantly following 2-Ads or 8-Ads compared to the mock-treated sample, with reduced MFIs against influenza A virus HAs, but not against influenza B viruses (Fig. 4). For A(H1N1) virus-infected persons (case A), post-infection MFIs against novel GH HA1 from H5, H7, and H9 were removed by 2-Ads (MFI < 1,000); MFIs against H3 HAs from strains earlier than A/Panama/2007/99 and MFIs against H5.Ind.05 E were reduced to <1,000 only after 8-Ads, although MFIs against all H1, H2, and early H3 HAs were still detectable (MFI > 1,500) (Fig. 4A). As shown in Fig. 4B [A(H1N1)pdm09], Fig. 4C [A(H3N2)], and Fig. 4D (influenza B virus), more complex antibody landscape shifts were observed for these serum samples, and MFIs against multiple Ecto and/or GH HA1 rHAs from H1, H2, H3, and H9 were still detectable (MFI ≥ 2,000) even after 8-Ads (Fig. 4B to D and Table S5).

### Analysis of correlation between HI/MN assays and MIADA.

**FIG 5:**
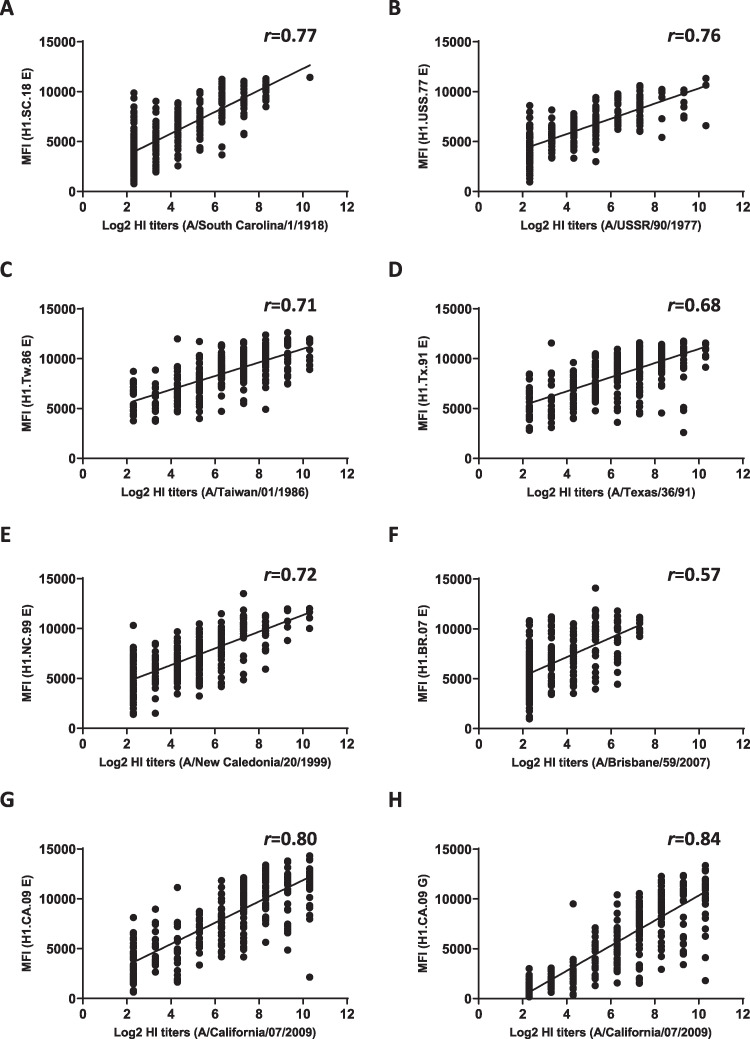
Correlation between HI titers and MFI values. A total of 162 paired serum samples collected from IIV recipients from 2010 to 2011, 2011 to 2012, 2013 to 2014, and 2016 to 2017 were tested by using six pre-2009 A(H1N1) viruses and one A(H1N1)pdm09 virus in the HI assay and the 43-plex MIADA. The Pearson correlation coefficient *r* values between log_2_ HI titers and MFI values against the corresponding HAs are plotted.

**FIG 6:**
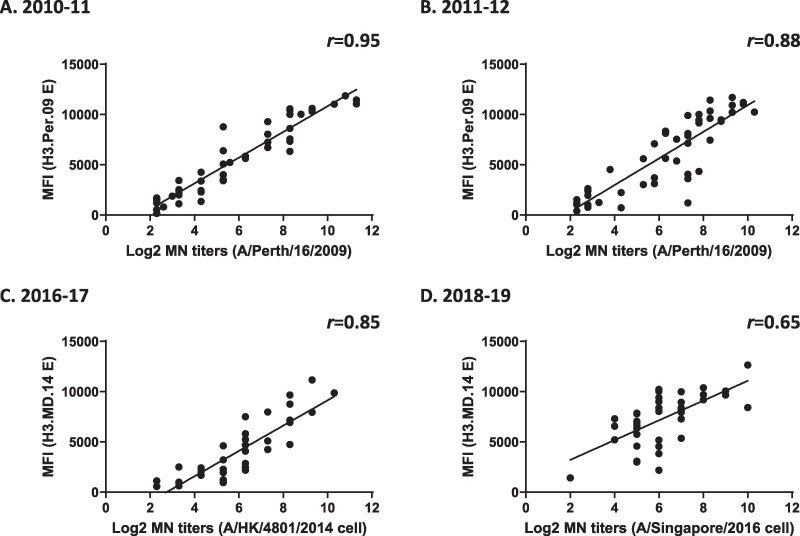
Correlation between MN titers and MFI values. Paired serum samples collected from IIV recipients from 2010 to 2018 were tested by using the MN assay and the 43-plex MIADA. Pearson correlation coefficient *r* values between log_2_-converted MN titers and MFI values against corresponding HAs were analyzed. (A) IIV, 2010 to 2011 (*n* = 23); (B) 2011 to 2012 (24 pairs); (C) 2016 to 2017 (19 pairs); (D) 2018 to 2019 (21 pairs).

We then analyzed the consistency of seroconversion between HI/MN assays and MIADA. In influenza virus serologic studies, a ≥4-fold rise in HI/MN titers is often considered a positive antibody response (or seroconversion). Here, in the MIADA, we used a ≥2-fold rise in MFIs (S2/S1) after adjustment of low MFIs in baseline S1 to 1,000 (to avoid an unreliable fold rise as described previously) as seroconversion (14, 15). We also analyzed consistency in non-seroconversion cases measured by HI (<4-fold rise) and MIADA (<2-fold rise). As shown in Table 5, we calculated the proportions of vaccinees who seroconverted by HI who also converted by MAIDA (seroconversion rate [SC]) and the proportion of HI nonconverters who nonconverted in MAIDA (non-seroconversion rate [NSC]); the SC and NSC in the MIADA ranged from 40% to 93% and from 73% to 100% for a total 162 paired samples from IIV recipients, respectively (Table 5). Similar sensitivities were achieved in HI/MN assays and MIADA for 17 influenza A or B virus-infected persons compared to the rRT-PCR results (Table 4). Finally, correlations between MFIs measured by MIADA and HI/MN titers were analyzed using 162 paired serum samples from IIV studies (Table 3). Pearson correlation coefficient *r* values between MFIs and HI titers against 8 A(H1N1) viruses ranged from 0.57 to 0.84 (Fig. 5), Pearson correlation coefficient *r* values between MFIs and MN titers against 3 A(H3N2) viruses ranged between 0.65 and 0.95 (Fig. 6). In summary, MFI titers measured by MIADA correlated well with HI/MN titers.

**TABLE 5:** Consistency in seroconversion/non-seroconversion between HI and MIADA

IIV yrs (no. of vaccinees)^*a*^	Rate (%)															
	A/SC/1/1918		A/USSR/90/77		A/Taiwan/1/86		A/Texas/36/91		A/NC/20/99		A/BR/59/2007		A/California/07/09 (E)		A/California/07/09 (G)	
	SC	NSC	SC	NSC	SC	NSC	SC	NSC	SC	NSC	SC	NSC	SC	NSC	SC	NSC
2010–2011 (49)	68^*b*^ (23^*d*^/34^*e*^)	60^*c*^ (9^*f*^/15^*g*^)	52 (14/27)	95 (21/22)	24 (4/17)	97 (31/32)	18 (3/17)	100 (32/32)	60 (3/5)	95 (42/44)	100 (3/3)	91 (42/46)	73^*h*^ (32/44)	100^*h*^ (5/5)	91^*i*^ (40/44)	80^*i*^ (4/5)
2011–2012 (58)	63 (22/35)	83 (19/23)	56 (10/18)	93 (37/40)	53 (9/17)	98 (40/41)	53 (9/17)	100 (41/41)	53 (6/11)	98 (46/47)	40 (2/5)	91 (48/53)	69 (29/42)	100 (16/16)	95 (40/42)	88 (14/16)
2013–2014 (27)	80 (8/10)	47 (8/17)	55 (6/11)	75 (12/16)	55 (6/11)	100 (16/16)	60 (6/10)	88 (15/17)	50 (3/6)	95 (20/21)	80 (4/5)	91 (20/22)	81 (22/27)	NA	93 (25/27)	NA
2016–2017 (28)	63 (5/8)	95 (19/20)	67 (2/3)	100 (25/25)	33 (1/3)	100 (25/25)	25 (1/4)	100 (24/24)	0 (0/4)	100 (24/24)	0 (0/3)	100 (25/25)	78 (7/9)	100 (19/19)	100 (9/9)	100 (19/19)

Total (162)	67 (58/87)	73 (55/75)	54 (32/59)	92 (95/103)	42 (20/48)	100 (25/25)	40 (19/48)	98 (112/114)	46 (12/26)	97 (132/136)	56 (9/16)	100 (25/25)	74 (90/122)	100 (40/40)	93 (114/122)	93 (37/40)

a IIV, inactivated influenza vaccine.

b SC, seroconversion rate in MIADA (percent).

c NSC, non-seroconversion rate in MIADA (percent).

d No. of SC by MIADA, MFI fold rise of ≥2.

e No. of SC by HI, HI fold rise of ≥4.

f No. of NSC by MIADA, MFI fold rise of <2.

g No. of NSC by HI, HI fold rise of <4.

h SC and NSC were calculated based on the fold rise in the MFI against H1.CA.09 E (ectodomain).

i SC and NSC were calculated based on fold rise in the MFI against H1.CA.09 G (globular head).

## DISCUSSION

In this study, we analyzed antibody landscapes following vaccination and infection using a high-throughput, serum-sparing (<10 μl sera needed per assay), 43-plex MIADA assay. Multiplex assays can be cost-effective, with higher throughput and fewer assay variations than the labor-intensive ELISA in detecting binding antibodies (6, 11, 14–16, 18).

Ferret antisera in HI assays have been used in influenza surveillance for decades for antigenic characterization of new emerging viruses. In our study, the MIADA assay using ferret antisera can detect the antigenic differences between HAs from influenza virus strains from inter-subtypes and/or intra-subtype (Table 2). To our knowledge, this is the first time that an extensive antigenic characterization against rHAs from multiple influenza viruses using ferret antisera has been reported in a multiplex binding assay platform. Recent studies showed limitations of using ferret antisera to detect influenza virus antigenic drift and certain antigenic changes of influenza viruses, such as egg adaptation (19–21). Therefore, human sera are needed for influenza surveillance and vaccine strain selections.

In human sera collected from vaccination and natural infection, significant antibody landscape shifts were detected (Fig. 1 to 3; see also Table S2 in the supplemental material). Vaccination and infection not only induced antibody responses against the exposed strains, but also back-boosted antibodies to the related viruses within the same subtype and even HAs from different subtypes, including novel subtype influenza viruses (Fig. 1 to 3 and Tables S2 to S5). Antibody landscapes showed clear age-related differences among three age groups both pre- and post-vaccination, a phenomenon that was also described by others (6, 8, 9). Previous studies also showed that antibodies to a novel HA(s) in unexposed populations can increase with age and may vary by geographical location (8, 9, 13, 15, 22–28). As expected, because adults are exposed to more seasonal influenza viruses and/or vaccines than young children, they have more complex antibody landscapes from past exposures through infection(s) and/or vaccination(s) with multiple influenza viruses (Fig. 2). Compared to young children, adults may also generate high levels of cross-reactive antibody responses. Both age and pre-vaccination antibody levels can affect influenza virus infection and vaccination responses (8, 9, 29).

It was suggested that natural infection may induce broader and longer-lasting antibody responses than vaccination (13, 30). Various antibody landscapes were observed in human sera collected from rRT-RCR-confirmed influenza A or influenza B virus-infected persons (Fig. 3). rRT-PCR is often considered the standard method for influenza virus detection, although viral RNA detection is limited to a short window of viral shedding. When well-paired serum samples are available, MIADA can also be used as a diagnostic method for both seasonal (Fig. 3) and novel influenza virus infections (14, 15). Our study showed that natural infections with A(H1N1)pdm09 and A(H3N2) viruses in some adults have induced highly cross-reactive binding antibodies against one or more novel subtype rHAs (Fig. 3 and Table S2); therefore, serum adsorption may become necessary for the serologic diagnosis of influenza virus infections, as we described previously (14, 15).

One of the first reports of cross-reactive antibodies against a novel influenza virus subtype was from primary A(H1N1) and A(H3N2) influenza virus-infected children, in whom cross-reactive antibodies to influenza H8 HA were detected by an ELISA, but not by HI (31). Other studies also showed that cross-reactive antibodies to both seasonal and novel influenza viruses exist in the population (11, 14, 15, 32). Cross-reactive antibodies may provide broader immune protection against influenza virus infections, including drifted and shifted influenza viruses. The assessment of cross-reactive antibodies to potentially pandemic strains is necessary for influenza pandemic preparedness (13, 15). Antibody landscape analysis using MIADA or other assays will be useful to assess the levels of immunity, including cross-reactive antibodies to novel viruses in populations.

While the HI assay measures the presence of antibodies that inhibit viral HA binding to host cells, MIADA was designed to detect total binding antibodies to Ecto and/or GH HA1. Although all serum samples were collected in the United States with no reported H5, H7, H9, and H13 human infections, cross-reactive antibodies against Ecto and/or GH HA1 from H5, H7, and H9 HAs were detected (Fig. 3C and Fig. 4). Since GH HA1 contains more subtype- and strain-specific epitopes (33), fewer cross-reactive antibodies against GH HA1 antigens were observed than against ectodomain rHAs (Fig. 3 and 4), and seroconversion detected by GH rHA1 in MIADA had a better correlation with HI seroconversion than that detected by ectodomain rHA (Table 5). Thus, in the context of serological diagnosis, the use of GH HA1 antigens in MIADA can improve the sensitivity and specificity for the detection of HA subtype-specific antibody responses (14, 15, 26). In contrast, to evaluate vaccine responses, paired ectodomain HA and HA1 or the future incorporation of stalk or chimera rHAs in the MIADA can be used to evaluate antibody responses against HA stalks that could be useful in HA stalk-targeting universal vaccine development.

To exclude the possibility of novel subtype influenza virus infection and to investigate the source of the cross-reactivity to novel influenza virus subtypes after exposure to seasonal strains, serum adsorption with 2 or 8 ectodomain rHAs from A(H1N1) and A(H3N2) was performed (14, 15). Antibodies against the novel subtype rHA(s) were completely removed by 2-Ads in three out of four infection cases that showed seroconversion in MFIs (Fig. 4 and Tables S3 and S4) and two out of nine S2 sera (Table S5), suggesting that these were cross-reactive antibodies to A(H1N1) and/or A(H3N2) viruses from past exposure rather than new infections with novel influenza viruses. Following 8-Ads, MFIs were reduced to less than 1,000 against the novel subtype HA(s), with the exception of H9N2 HA (H9.HK.09 G); interestingly, MFI values against H2 and H9 were correlated in 4 out of 9 S2 sera (Fig. 4 and Table S5). Stephenson et al. reported that individuals born before 1969 showed 31% and 83% MN titers against A(H9N1) and A(H9N2) viruses, and H9 baseline reactivity was related to A(H2N2), but not to A(H1N1) and A(H3N2) viruses (34). All infected persons in this study were born before 1967, who were likely exposed to A(H2N2) viruses. It is interesting to note that here, MFIs against H9 HA were not always correlated with MFIs against H2 HA (Table S5). Following 8-Ads, S2 serum from A(H1N1)pdm09 infection (case A) lost binding to H9, while MFIs against H2 remained (Fig. 4A), and 4 out of 9 S2 sera from persons who did not show seroconversion showed MFIs of >2,000 for both H2 and H9 HAs (Fig. 4 and Table S5). Our results suggested that cross-reactive antibodies against novel subtype HA in some persons might be generated from previous exposure to pre-2009 A(H1N1), A(H3N2), or even A(H2N2) viruses.

Antibody responses detected by MIADA correlated well with HI and MN titers in sera collected from both influenza vaccination and infection (Fig. 5 and 6 and Tables 3 and 5); this is consistent with our previous reports (14–16, 18). Other studies also showed that MN/HI titers correlated with binding antibodies detected by protein microarrays (6, 11, 13). Multiplex protein microarrays (11, 35, 36) and other magnetic fluorescence microsphere immunoassays (16) using rHAs can also provide a high-throughput alternative to replace traditional ELISAs. However, these assays differ in antigen conjugation, presentation (for example, dried antigens spotted on microarray slides versus antigens conjugated on a microsphere in liquid), and antigen numbers that may lead to differences in assay performance, which warrants further evaluation.

Our study has several limitations. First, in the current 43-plex assay, we were not able to include recombinant HA stalk antigens and other influenza virus surface antigens such as neuraminidase, which could offer a broader scope of response in the antibody landscape analysis; second, sample sizes from the influenza virus infection cases were small, which limited our ability to evaluate additional factors (such as age and prior vaccination) that could impact antibody landscape changes following influenza virus infections.

In summary, MIADA combined with serum adsorption with ectodomain H1/H3 rHAs offered advantages in analysis of antibody landscapes, confirmation of the origin of cross-reactive antibodies, and detection of HA subtype-specific antibody responses. MIADA will be a valuable, rapid, high-throughput tool to aid vaccine development and elucidate the complex human immunity to influenza virus.

## MATERIALS AND METHODS

### Human and ferret sera.

A total of 227 paired blood specimens (pre-vaccination [S1] and post-vaccination [S2]) were collected from inactivated influenza vaccine (IIV) recipients from 5 influenza seasons (Table 3). All sera were tested against virus strains that were included in seasonal IIVs (Table 3) as well as 7 viruses (A/South Carolina/1/1918, A/USSR/90/77, A/Taiwan/1/86, A/Texas/36/91, A/New Caledonia/20/99, A/Brisbane/59/2007, and A/California/07/2009) by the HI assay. Acute (S1)- and convalescent (S2)-phase sera, where possible, were collected from influenza virus-positive veterans enrolled in the Surveillance Platform for Enteric and Respiratory Infectious Organisms at the VA (SUPERNOVA) at the Michael E. DeBakey VA Medical Center in Houston, TX, and the West Los Angeles VA Medical Center in Los Angeles, CA. SUPERNOVA conducted active enrollment of veterans who were hospitalized due to acute respiratory illness (ARI) symptoms, and multiplex respiratory pathogen testing by real-time reverse transcription-PCR (rRT-PCR) was completed at each site. Influenza virus-positive cases verified by rRT-PCR were eligible for acute- and convalescent-phase serum collection. A total of 17 paired human sera (S1 and S2) were collected from rRT-PCR-confirmed influenza A(H1N1)pdm09 (*n* = 2), influenza A(H3N2) (*n* = 10), and influenza B virus-infected persons (*n* = 5) (Table 4). All sera were tested for A(H1N1), A(H3N2), or influenza B viruses by HI and/or MN assays. The use of human sera was approved by National Centers for Immunization and Respiratory Diseases, Centers for Disease Control and Prevention Research Determination Review.

Ferret antisera were generated using either primary infection with influenza virus or immunization with rHAs (see Table S1 in the supplemental material).

### High-throughput multiplex influenza antibody detection assay.

The multiplex influenza antibody detection assay (MIADA) was developed by using 42 trimeric ectodomain and/or GH HA1 antigens from influenza A viruses, including pre-2009 A(H1N1), A(H1N1)pdm09, A(H2N2), A(H3N2), A(H5N1), A(H7N7), A(H7N9), A(H7N2), A(H9N2), and A(H13N9); influenza B/Brisbane/60/2008 (B Victoria lineage) and B/Wisconsin/1/2010 (B Yamagata lineage) viruses; and a protein A (PA) control (Table 1). The HA antigens were either obtained from the International Reagent Resource (IRR) (https://www.internationalreagentresource.org/About/IRR.aspx) or expressed and purified using an in-house baculovirus expression system (37–39). We first optimized the best ratio of HA amounts (micrograms) and bead numbers and then adjusted the antigen amount based on the molecular mass of each antigen to keep the same molar ratio of each antigen to bead number. As described previously, 100 μg of ectodomain, 60 μg of each GH HA1, or 22 μg of PA was coupled to 6.25 × 10^6^ Bio-Plex Pro magnetic COOH beads (Bio-Rad, CA) (15). Fifty microliters of microspheres containing 2,000 microspheres for each of 43 bead regions in assay buffer was added to each well of a black-wall plate (86,000 microspheres/well), followed by the incubation of 1:40-diluted human serum samples or a pre-determined optimal dilution of ferret sera in duplicates (Table S1), and two serum pools were included on each plate as intra- and inter-assay controls. After a wash with assay buffer, a R-phycoerythrin‐conjugated protein A (RPE-PA) reporter was added, followed by incubation. MFIs were obtained by a Bio-Plex Magpix multiplex reader as described previously (15). If the differences of MFIs in duplicates were >20%, the samples were retested. MFI values, delta values (S2 value − S1 value), and fold rises in MFIs (S2 value/S1 value) were analyzed to measure antibody binding. Given the wide dynamic ranges of the readout of the MIADA, S1 samples with MFI values of <1,000 were adjusted to 1,000 to achieve reliable fold rise results (≥2-fold rise in MFIs as seroconversion in MIADA), as described previously (14, 15).

### Serum antibody adsorption with mock adsorption, two-rHA adsorption, or eight-rHA adsorption.

Serum adsorption was performed with latex beads conjugated with two ectodomain rHAs (2-Ads), or with a cocktail of nickel-coated magnetic beads bound with eight rHAs (8-Ads), as described previously (14, 15, 40); mock adsorption with beads only was also performed as a control.

### HI and MN assays.

The HI assay was performed as described previously (41); briefly, nonspecific inhibitors in the sera were removed by incubation with receptor-destroying enzyme at 37°C for 18 to 20 h, followed by heat inactivation at 56°C for 30 min. Serially 2-fold-diluted sera were tested in duplicate using 0.5% turkey red blood cells. MN assays were performed as described previously (41). Sera were heat inactivated at 56°C for 30 min, and serial 2-fold dilutions were then mixed with influenza viruses (100 50% tissue culture infective doses [TCID_50_]). The mixtures were incubated at 37°C with 5% CO_2_ for 1 h, followed by infecting 1.5 × 10^4^ Madin-Darby canine kidney-SIAT1 (MDCK-SIAT1) cells per well of a 96-well plate. After an 18-h incubation at 37°C with 5% CO_2_, viral infection was determined by an ELISA using a mouse anti-influenza virus A nucleoprotein (NP) monoclonal antibody pool (A1 and A3; Millipore, CA). Neutralizing antibody titers were defined as the reciprocal of the highest dilution of serum samples that achieved at least 50% neutralization (41).

### Statistical analysis.

Mean MFIs and 95% confidence intervals (CIs) were calculated. Seroconversion by MFI is defined as a ≥2-fold rise from S1 to S2 with an S1 MFI of <1,000 adjusted to 1,000. Seroconversion by HI or MN is defined as a 4-fold rise from S1 to S2 with S2 titers of ≥40. Antibody responses between the seasons or between age groups were compared using either one-way analysis of variance (ANOVA) or unpaired two-tailed *t* tests. Antibody responses pre- and post-vaccination were compared using two-tailed paired *t* tests. The correlation coefficient *r* values were calculated using Pearson correlation analysis. Statistical analysis was performed using GraphPad Prism 8.
