# Supplementary material for: Antibody Landscape Analysis following Influenza Vaccination and Natural Infection in Humans with a High-Throughput Multiplex Influenza Antibody Detection Assay
Source: mBio. 2021 Feb 2;12(1):e02808-20. doi: 10.1128/mBio.02808-20 (PMC7858056; doi:10.1128/mBio.02808-20)
Supplement: FIG S1 [file mBio.02808-20-sf001.pdf]

A. S1

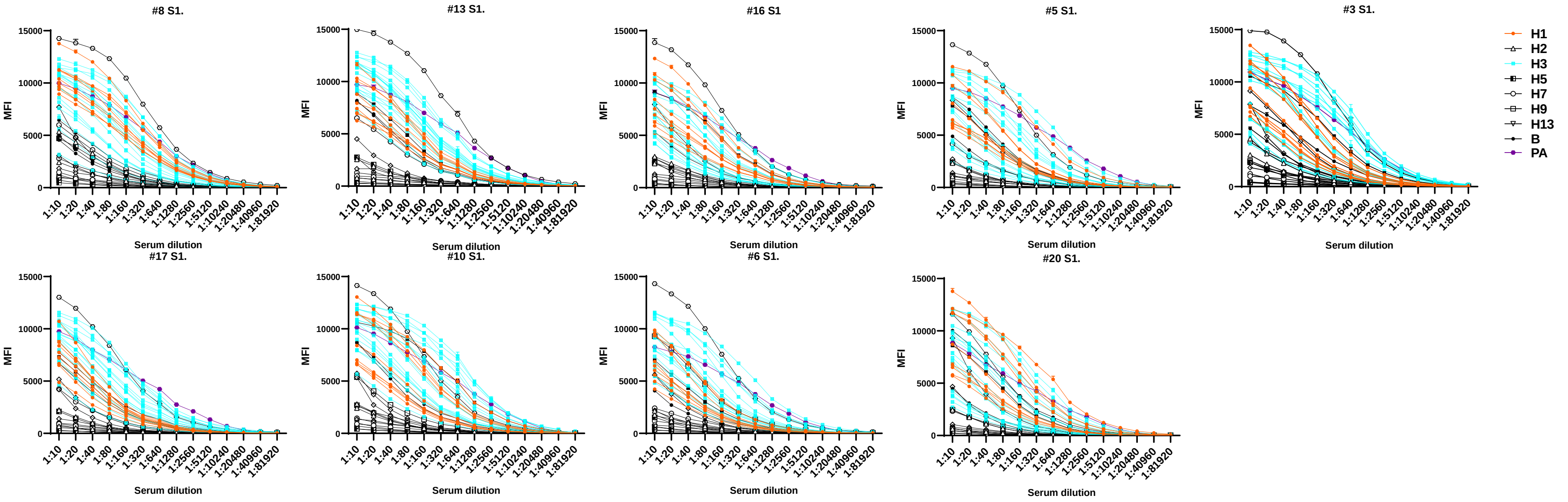

B. S2

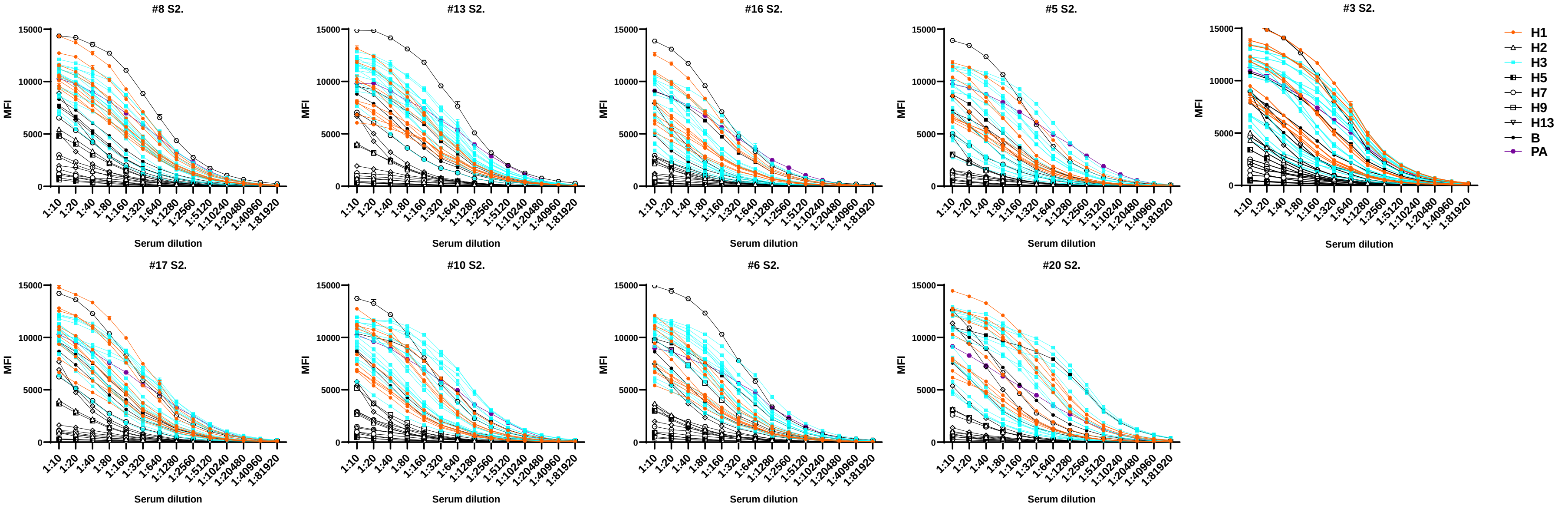

FIG S1 Linearity of the MIADA assay. A total of 9 paired sera from vaccine recipients in 2018-19 were tested in duplicates at serial 2-fold dilutions starting from 1:10 to 1:81920 by the multiplex MIADA assay. (A) Pre-vaccination (S1) sera; (B). Post-vaccination (S2) sera. Responses to virus HA antigens are color coded by subtypes: H1, H2, H3, H5, H7, H9, H13, influenza B, and protein A (PA).
