# Supplementary material for: Antibody Landscape Analysis following Influenza Vaccination and Natural Infection in Humans with a High-Throughput Multiplex Influenza Antibody Detection Assay
Source: mBio. 2021 Feb 2;12(1):e02808-20. doi: 10.1128/mBio.02808-20 (PMC7858056; doi:10.1128/mBio.02808-20)
Supplement: FIG S2 [file mBio.02808-20-sf002.pdf]

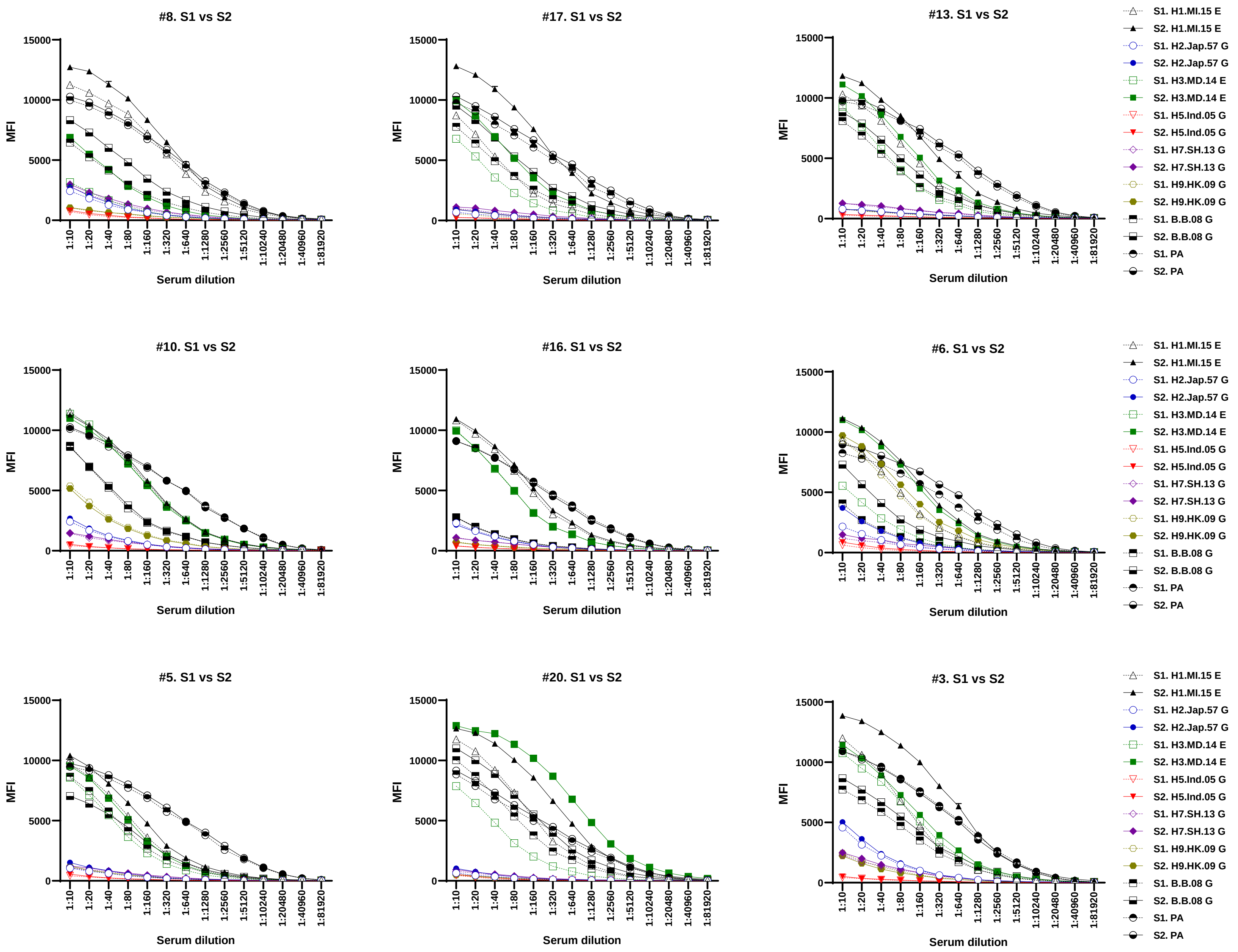

FIG S2 Linear range of MFIs in MIADA assay pre- and post-vaccination. A total of 9 paired sera from vaccine recipients in 2018-19 were tested in duplicates at serial 2-fold dilution starting from 1:10 to 1:81920 by the MIADA assay. MFI pre-(S1) and post-vaccination (S2) to 8 antigens were plotted together for each vaccine recipient to demonstrate linear range of the responses. PA: protein A.
