# Supplementary material for: Antibody Landscape Analysis following Influenza Vaccination and Natural Infection in Humans with a High-Throughput Multiplex Influenza Antibody Detection Assay
Source: mBio. 2021 Feb 2;12(1):e02808-20. doi: 10.1128/mBio.02808-20 (PMC7858056; doi:10.1128/mBio.02808-20)
Supplement: FIG S3 [file mBio.02808-20-sf003.pdf]

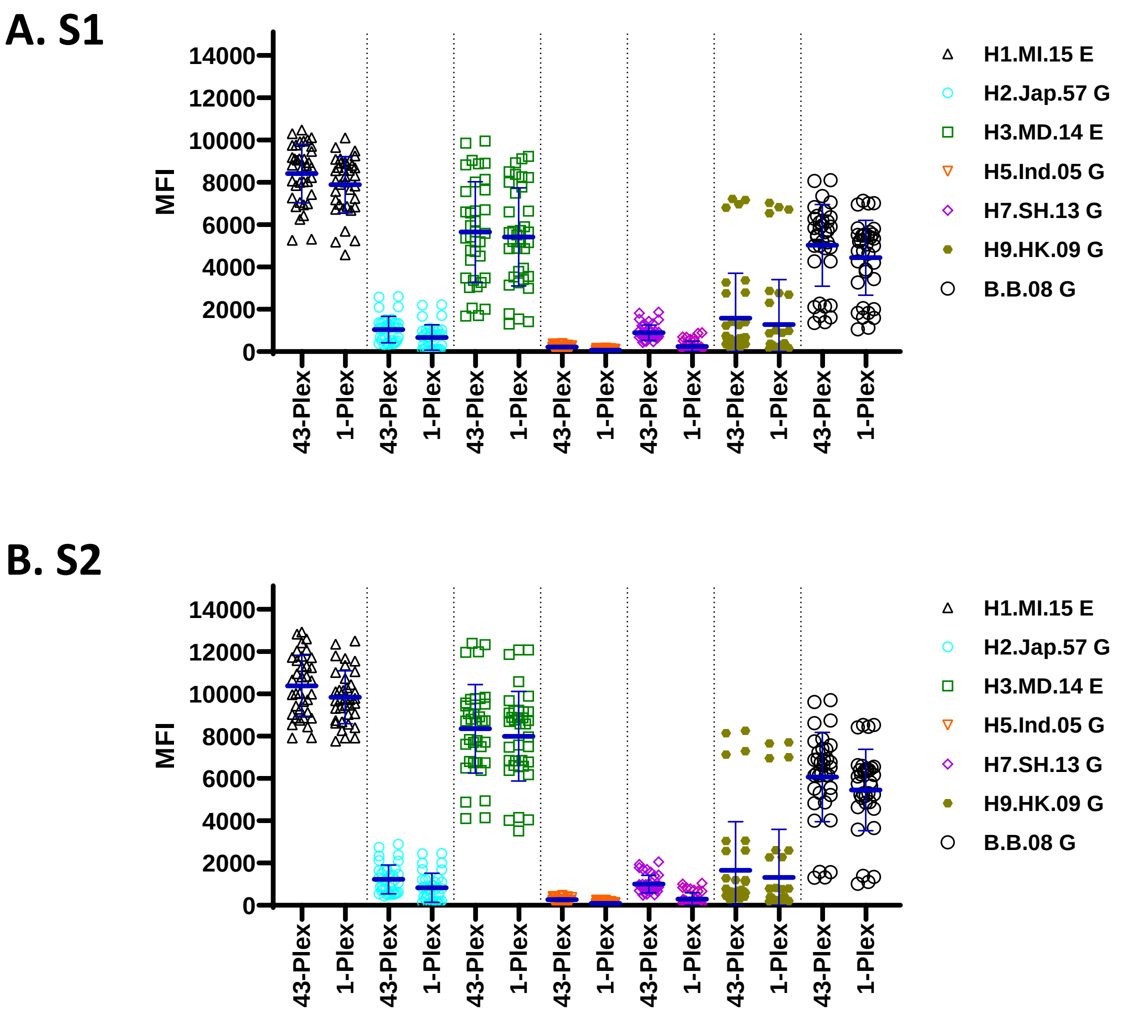

FIG S3 Comparison of MFI values between 43-Plex and 1-Plex MIADA assay. A total 9 paired sera from vaccine recipients in 2018-19 were tested in duplicates at 1:40 dilution by the MIADA assay. The mean and standard deviation of MFIs to seven representative antigens from two independent experiments were plotted. A. S1 (pre-vaccination); B. S2 (post-vaccination).
