# Supplementary material for: Antibody Landscape Analysis following Influenza Vaccination and Natural Infection in Humans with a High-Throughput Multiplex Influenza Antibody Detection Assay
Source: mBio. 2021 Feb 2;12(1):e02808-20. doi: 10.1128/mBio.02808-20 (PMC7858056; doi:10.1128/mBio.02808-20)
Supplement: TABLE S1 [file mBio.02808-20-st001.pdf]

TABLE S1 Ferret antisera used in this study. Details of the ferret sera used in antigenic characterization (Table 2) are listed.

Ferret antisera raised against	Abbreviation	Immunization method	IRR Cat [2]	source	Dilutions [3]
NA	NFS[1]	NA	FR-282	IRR	1:80
A/Marton/1943	FαH1/Mar	rHA	FR-951	IRR	1:160
A/USSR/90/1977	FαH1/USS	rHA	FR-953	IRR	1:80
A/Taiwan/01/1986	FαH1/Tw	rHA	FR-954	IRR	1:80
A/Texas/36/1991	FαH1/Tx	Infection	NA	CDC	1:80
A/New Caledonia/20/1999	FαH1/NC	rHA	FR-955	IRR	1:160
A/Brisbane/59/2007	FαH1/BR	rHA	FR-388	IRR	1:80
A/California/7/2009	FαH1/CA	rHA	FR-359	IRR	1:640
A/Michigan/45/2015	FαH1/MI	Infection	NA	CDC	1:80
A/Japan/305/1957	FαH2/Jap	rHA	FR-891	IRR	1:640
A/Hong Kong/8/1968	FαH3/HK	Infection	NA	CDC	1:640
A/Victoria/3/1975	FαH3/VC	Infection	NA	CDC	1:10240
A/Bangkok/1/1979	FαH3/BK	Infection	NA	CDC	1:80
A/Shanghai/11/1987	FαH3/SH	Infection	NA	CDC	1:1280
A/Beijing/32/1992	FαH3/BJ	Infection	NA	CDC	1:640
A/Sydney/5/1997	FαH3/Syd	Infection	NA	CDC	1:640
A/Fujian/411/2002	FαH3/FJ	Infection	NA	CDC	1:80
A/Perth/16/2009	FαH3/Per	Infection	NA	CDC	1:640
A/Victoria/361/2011	FαH3/VC	Infection	NA	CDC	1:640
A/Texas/50/2012	FαH3/Tx	Infection	NA	CDC	1:1280
A/Switzerland/9715293/2013	FαH3/SW	Infection	NA	CDC	1:320
A/Vietnam/1203/2004	FαH5/VN	rHA	FR-708	IRR	1:2560
A/Indonesia/05/2005	FαH5/Ind	Infection	NA	CDC	1:80
A/Netherlands/219/2003	FαH7/NED	rHA	FR-890	IRR	1:640
A/Shanghai/2/2013	FαH7/SH	rHA	FR-1280	IRR	1:640
A/New York/108/2016	FαH7/NY	Infection	NA	CDC	1:640
A/Hong Kong/33982/2009	FαH9/HK	rHA	FR-1084	IRR	1:640
A/Shorebird/DE/68/2004	FαH13/DE	rHA	FR-290	IRR	1:2560
B/Brisbane/60/2008	FαB/BR	rHA	FR-392	IRR	1:80
B/Wisconsin/1/2010	FαB/Wis	rHA	FR-810	IRR	1:80

1.normal ferret sera

2. IRR-International Reagent Resource.

3. Dilutions used in Table 2. MFI values against PA control in table correlates with the ferret sera dilutions used.

CDC: centers for disease control and prevention.
