# Supplementary material for: Antibody Landscape Analysis following Influenza Vaccination and Natural Infection in Humans with a High-Throughput Multiplex Influenza Antibody Detection Assay
Source: mBio. 2021 Feb 2;12(1):e02808-20. doi: 10.1128/mBio.02808-20 (PMC7858056; doi:10.1128/mBio.02808-20)
Supplement: TABLE S2 [file mBio.02808-20-st002.pdf]

Table S2. MFI delta values between pre- (S1) and post-vaccination (S2) in 2010-2018 and pre- (S1) and post-infection (S2) in 2016-2018 seasons. Delta values = S2-S1. Color scale indicates MFI levels

Antigen	IIV 2010-11*		IIV 2011-12		IIV 2013-14		IIV 2016-17		IIV 2018-19		IIV 2018-19		IIV 2018-19		Infection during 2016-18**						
									< 3 yrs		9-17 yrs		19-49 yrs		Flu B		A(H3N2)		A(H1N1)		
	Mean	95% CI	Mean	95% CI	Mean	95% CI	Mean	95% CI	Mean	95% CI	Mean	95% CI	Mean	95% CI	Mean	95% CI	Mean	95% CI	Mean	95% CI	color scale
H1.SC.18 E	3500	2862-4138	3156	2573-3740	3618	2877-4358	2252	1418-3087	3196	1817-4575	4459	3188-5730	2790	1571-4009	104	-332-541	741	70-1412	4458	N/A***	50
H1.PR.34 E	1568	1246-1891	1491	1169-1813	1493	1065-1922	953	658-1249	2138	1060-3215	1489	1007-1971	1412	702-2122	238	-297-774	716	89-1343	3736	N/A	100
H1.Mar.43 E	1849	1398-2299	1506	1087-1926	2039	1523-2556	1054	734-1374	3474	2017-4931	2263	1788-2738	1482	657-2308	0	-216-216	465	14-916	4309	N/A	200
H1.USS.77 E	1826	1464-2189	1428	948-1909	1842	1304-2380	1032	621-1443	2953	1583-4323	1627	1296-1958	1180	546-1815	-10	-314-294.6	265	-86-616	3789	N/A	400
H1.Tw.86 E	1505	1119-1892	1492	1045-1940	1832	1428-2236	1063	697-1429	3499	1978-5020	2389	1830-2947	1640	802-2478	82	-416-580.6	843	132-1554	2828	N/A	600
H1.Tx.91 E	907	543-1271	1024	595-1453	1695	1116-2275	711	402-1020	3377	1863-4891	2398	1753-3043	1245	428-2061	-38	-409-333	384	-197-965	2546	N/A	800
H1.NC.99 E	1395	1017-1773	1334	873-1795	1687	1202-2173	810	489-1131	2877	1459-4295	1635	1177-2093	762	182-1342	16	-459-491	587	47-1127	3333	N/A	1000
H1.BR.07 E	1726	1288-2165	1436	1003-1870	2122	1551-2694	891	544-1240	2979	1532-4427	1820	1316-2323	915	241-1590	74	-500-648	687	110-1264	3876	N/A	1500
H1.CA.09 E	4873	4200-5546	4364	3705-5022	5595	4848-6343	2881	1840-3923	6196	4333-8060	5211	3667-6755	3000	1497-4504	155	-469-779	454	43-865	5507	N/A	2000
H1.CA.09 G	5961	5150-6772	5176	4368-5984	5914	5004-6823	3134	1910-4359	4880	3149-6611	5005	3400-6611	3291	1688-4895	297	-454-1048	705	67-1343	6330	N/A	2500
H1.MI.15 E	3389	2845-3933	3252	2715-3790	4586	3952-5221	2444	1725-3163	6664	5315-8013	5208	4003-6414	2950	1667-4233	707	-283-1697	295	-101-691	4290	N/A	3000
H2.Jap.57 G	1251	831-1670	910	633-1188	562	301-824	423	1889-658	353	204-503	226	159-293	463	205-720	87	-262-435	1029	216-1843	934	N/A	3500
H3.HK.68 E	663	496-831	769	372-1167	1022	627-1418	743	461-1025	1899	1109-2688	897	675-1120	823	498-1148	338	-193-869	1427	480-2374	762	N/A	4000
H3.PC.73 E	548	373-722	766	324-1209	843	534-1153	516	275-757	1683	1052-2313	779	573-986	737	445-1030	504	-601-1609	1481	468-2494	998	N/A	4500
H3.VC.75 E	662	410-913	1054	497-1612	1146	710-1582	726	444-1008	3020	1736-4304	845	615-1075	1033	490-1577	269	-474-1013	1332	315-2350	816	N/A	5000
H3.BK.79 E	410	229-592	827	412-1243	904	464-1345	441	215-667	2035	846-3224	453	328-578	454	183-726	765	-493-2022	981	403-1560	859	N/A	6000
H3.LN.86 E	525	249-802	1112	628-1596	1048	556-1540	605	277-933	2403	1137-3669	639	428-849	713	55-1372	349	-738-1435	1112	398-1826	340	N/A	7000
H3.SH.87 E	494	244-744	960	568-1352	903	368-1437	823	487-1158	1475	523-2427	908	476-1339	684	114-1254	328	-607-1264	1326	509-2142	562	N/A	
H3.BJ.92 E	756	319-1193	1123	670-1576	1262	703-1820	998	623-1374	2389	1108-3669	1077	607-1548	1087	454-1720	96	-587-778	1785	805-2765	1266	N/A	
H3.JH.94 E	740	290-1191	992	521-1464	1087	455-1720	828	466-1189	1403	560-2246	701	339-1064	592	269-916	-86	-375-203	1674	388-2960	606	N/A	
H3.NC.95 E	1283	810-1756	1730	1259-2201	1917	1064-2771	1375	917-1832	1499	704-2294	644	439-849	803	337-1270	190	-444-824	1782	865-2699	642	N/A	
H3.Syd.97 E	1010	652-1368	1132	706-1557	1107	716-1499	1079	658-1499	2092	1091-3094	2019	1373-2665	539	296-782	59	-365-483	1525	570-2481	911	N/A	
H3.Pan.99 E	1515	1036-1995	1715	1172-2257	1557	924-2191	1654	998-2310	2773	1446-4101	2573	1723-3424	733	335-1132	30	-309-369	1789	392-3185	910	N/A	
H3.WY.03 E	1899	1398-2400	2195	1653-2737	2024	1349-2700	1925	1231-2619	3301	2031-4570	2781	2003-3558	808	415-1200	70	-543-683.5	1526	684-2367	962	N/A	
H3.CA.04 E	2612	2052-3173	2512	1982-3042	2389	1663-3115	2076	1481-2670	2984	1807-4161	2261	1625-2897	1009	603-1416	169	-417-756	1434	541-2326	601	N/A	
H3.WI.05 G	2387	1928-2847	2258	1818-2697	2199	1696-2702	1750	1204-2296	3699	2464-4934	1737	1204-2270	964	480-1448	25	-327-377	2690	1114-4267	1588	N/A	
H3.Per.09 E	4351	3524-5178	4429	3701-5157	4026	3126-4926	3549	2475-4623	5936	4775-7098	2566	1721-3411	1996	966-3026	-18	-450-415	2889	1416-4362	690	N/A	
H3.Per.09 G	3518	2903-4133	3633	3062-4204	3152	2488-3817	2856	1990-3721	4907	3721-6092	1793	1123-2463	1524	764-2284	-74	-307-159	3214	1504-4923	1797	N/A	
H3.VC.11 E	4177	3389-4965	4168	3474-4862	4148	3299-4998	3701	2679-4724	6114	4988-7240	2849	2090-3609	2257	1273-3242	41	-411-494	2598	1205-3990	642	N/A	
H3.Tx.12 E	4340	3542-5138	4546	3812-5280	4452	3556-5348	3755	2662-4848	6442	5232-7652	2736	1878-3594	2218	1169-3266	-61	-573-450	2770	1303-4237	855	N/A	
H3.SW.13 E	1656	1188-2124	1911	1492-2331	2412	1601-3223	3784	2808-4760	5220	3986-6454	3753	2973-4532	3306	2244-4368	208	-280-696	2166	843-3490	558	N/A	
H3.MD.14 E	1941	1450-2433	2231	1751-2712	2589	1829-3349	3225	2419-4031	4917	3674-6160	3586	2677-4495	3114	2044-4183	338	-68-744	2692	1215-4168	393	N/A	
H5.VN.04 G	208	97-319	201	115-286	104	2-206	87	34-140	59	16-102	60	11-111	62	-10-134	17	-152-186	461	-102-1023	217	N/A	
H5.Ind.05 E	711	501-921	741	562-920	1135	807-1464	668	458-879	1226	685-1767	780	544-1016	884	527-1242	81	-262-424	381	101-660	2509	N/A	
H5.Ind.05 G	293	104-481	266	142-391	141	63-219	126	61-191	65	11-120	25	-36-87	80	-31-190.8	38	-104-181	514	-110-1137	674	N/A	
H7.NED.03 G	317	157-477	245	156-334	189	70-309	109	41-178	67	14-120	19	10-28	112	30-194	16	-40-72	379	99-659	946	N/A	
H7.SH.13 G	440	245-636	339	220-459	275	142-409	180	97-265	177	79-275	94	65-124	201	80-322	1	-85-87	528	146-909	665	N/A	
H7.NY.16 E	319	230-408	337	235-439	561	317-806	500	301-700	525	215-835	415	217-613	475	272-677	125	-72-322	457	160-753	1396	N/A	
H9.HK.09 G	629	190-1067	211	119-304	397	-22-817	318	77-559	407	-50-864	63	-16-143	116	23-209	171	-301-642	813	-36-1663	101	N/A	
H13.DE.04 G	167	27-308	33	16-51	37	4-70	47	-4-97	248	76-421	86	-9-182	82	8-156	4	-12-21	44	19-68	5	N/A	
B.B.08 G	2372	1707-3037	3383	2717-4049	2300	1453-3148	2290	1582-2999	2877	1798-3956	2747	1833-3661	2519	1536-3502	2611	-1095-6318	-92	-307-123	-139	N/A	
B.W.10 G	802	478-1127	1182	850-1515	2773	1986-3560	1931	1257-2605	3449	2515-4382	3580	2835-4325	2475	1623-3328	3132	-340-6604	-37	-169-95	-123	N/A	
PA	50	9-91	80	0-160	95	30-160	93	41-145	-107	-350-136	105	28-183	11	-66-88.97	-315	-1308-678	50	-32-132	307	N/A	

*IIV samples from five influenza seasons during 2010-2018
**infected persons during 2016-2018 influenza seasons
***N/A: not applicable
All numbers represented delta values of MFIs.
