# Supplementary material for: Antibody Landscape Analysis following Influenza Vaccination and Natural Infection in Humans with a High-Throughput Multiplex Influenza Antibody Detection Assay
Source: mBio. 2021 Feb 2;12(1):e02808-20. doi: 10.1128/mBio.02808-20 (PMC7858056; doi:10.1128/mBio.02808-20)
Supplement: TABLE S3 [file mBio.02808-20-st003.pdf]

TABLE S3 MFI values against novel subtype HAs after mock or 2-Ads among A(H1N1)pdm09 and A(H3N2) infection cases with MFI seroconversion to novel subtype influenza viruses. Color scale indicates MFI levels.

Serum treatment	Case ID-S1/S2	Infected by	H2.Jap.57 G	H5.VN.04 G	H5.Ind.05 E	H5.Ind.05 G	H7.NED.03 G	H7.SH.13 G	H7.NY.16 E	H9.HK.09 G	H13.DE.04 G
**Mock**	**A-S1**	**A(H1N1)pdm09**	**5871**	228	969	433	162	227	488	372	323
	**A-S2**	**A(H1N1)pdm09**	**7413**	281	**5224**	1539	1720	1376	**2957**	502	355
	**B-S1**	**A(H3N2)**	**6125**	161	1844	111	66	116	457	827	59
	**B-S2**	**A(H3N2)**	**8381**	1029	**2181**	209	203	339	845	**4418**	84
	**C-S1**	**A(H3N2)**	**4993**	347	**3709**	415	217	360	547	829	77
	**C-S2**	**A(H3N2)**	**6860**	**2390**	**4234**	1752	1201	1662	1518	**2409**	149
	**D-S1**	**A(H3N2)**	**2527**	375	859	688	133	166	772	850	106
	**D-S2**	**A(H3N2)**	**5375**	1034	1898	**2794**	931	1463	1356	1005	171
**2-Ads**	**A-S1**	**A(H1N1)pdm09**	**5901**	203	402	232	62	89	339	302	286
	**A-S2**	**A(H1N1)pdm09**	**6183**	236	**4770**	298	109	163	1025	397	296
	**B-S1**	**A(H3N2)**	**5518**	153	165	107	55	80	277	556	53
	**B-S2**	**A(H3N2)**	**6476**	167	251	95	67	112	264	754	57
	**C-S1**	**A(H3N2)**	**4653**	157	1270	184	72	122	371	447	67
	**C-S2**	**A(H3N2)**	**5047**	674	1990	216	66	157	1010	600	80
	**D-S1**	**A(H3N2)**	**2265**	328	275	552	61	70	546	825	89
	**D-S2**	**A(H3N2)**	**2895**	443	515	1099	145	191	690	920	96

Color scale
50
100
200
400
600
800
1000
1500
2000
3000
4000
5000
6000
7000
8000
9000
10000

Cross-reactive antibody responses to novel subtype HAs in three out of four patients were induced by exposures to A(H1N1)pdm09 (A/California/07/2009) and/or A/Perth/16/2009 like HAs.
MFIs against H2, H5, H7, H9, and H13 HA higher than 2000 were highlighted in bold.
