# Supplementary material for: Antibody Landscape Analysis following Influenza Vaccination and Natural Infection in Humans with a High-Throughput Multiplex Influenza Antibody Detection Assay
Source: mBio. 2021 Feb 2;12(1):e02808-20. doi: 10.1128/mBio.02808-20 (PMC7858056; doi:10.1128/mBio.02808-20)
Supplement: TABLE S4 [file mBio.02808-20-st004.pdf]

TABLE S4 Fold rise after mock or 2-Ads among A(H1N1)pdm09 and A(H3N2) infection cases with MFI seroconversion to novel subtype influenza viruses. Color scale indicates fold rise levels.

Case ID-treatment	Infected by	H2.Jap.57 G	H5.VN.04 G	H5.Ind.05 E	H5.Ind.05 G	H7.NED.03 G	H7.SH.13 G	H7.NY.16 E	H9.HK.09 G	H13.DE.04 G
A-Mock	A(H1N1)pdm09	1.26	0.28	**5.22**	1.54	1.72	1.38	**2.96**	0.50	0.36
A-2-Ads	A(H1N1)pdm09	1.05	0.24	**4.77**	0.30	0.11	0.16	1.03	0.40	0.30
B-Mock	A(H3N2)	1.37	1.03	1.18	0.21	0.20	0.34	0.84	**4.42**	0.08
B-2-Ads	A(H3N2)	1.17	0.17	0.25	0.10	0.07	0.11	0.26	0.75	0.06
C-Mock	A(H3N2)	1.37	**2.39**	1.14	1.75	1.20	1.66	1.52	**2.41**	0.15
C-2-Ads	A(H3N2)	1.08	0.67	1.57	0.22	0.07	0.16	1.01	0.60	0.08
D-Mock	A(H3N2)	**2.13**	1.03	1.90	**2.79**	0.93	1.46	1.36	1.01	0.17
D-2-Ads	A(H3N2)	1.28	0.44	0.52	1.10	0.14	0.19	0.69	0.92	0.10

Color scale
0.1
0.2
0.5
1.0
**2.0**
**4.0**
**5.0**
**6.0**

Fold rises in MFIs > 2-fold were highlighted in bold.
Cross reactive antibody responses to novel subtype HAs in three out of four patients were induced by exposures to A(H1N1)pdm09 (A/California/07/2009) and/or A/Perth/16/2009 like HAs.
