# Supplementary material for: Antibody Landscape Analysis following Influenza Vaccination and Natural Infection in Humans with a High-Throughput Multiplex Influenza Antibody Detection Assay
Source: mBio. 2021 Feb 2;12(1):e02808-20. doi: 10.1128/mBio.02808-20 (PMC7858056; doi:10.1128/mBio.02808-20)
Supplement: TABLE S5 [file mBio.02808-20-st005.pdf]

TABLE S5. MFIs against novel subtype Ecto and/or GH HA1 detected in 9 S2 sera were partially removed by 2-Ads or 8-Ads.
Color scale indicates MFI values.

Mock

Case ID	Infected by	H2.Jap.57 G	H5.VN.04 G	H5.Ind.05 E	H5.Ind.05 G	H7.NED.03 G	H7.SH.13 G	H7.NY.16 E	H9.HK.09 G	H13.DE.04 G
E	A(H1N1)pdm09	**7442**	1978	**3479**	1078	710	661	642	**3780**	266
F	A(H3N2)	**2648**	1334	**3050**	1589	639	1089	622	1189	136
G	A(H3N2)	708	126	1392	97	250	359	**3495**	260	150
H	A(H3N2)	**9279**	347	**2267**	1216	1158	1645	1631	**2948**	203
I	A(H3N2)	**5988**	580	**3514**	752	171	398	594	**5088**	159
J	A(H3N2)	**5452**	**2367**	**2236**	**2732**	387	677	1009	**4512**	510
K	A(H3N2)	**5356**	755	**3312**	376	419	572	1493	**247**	**2234**
L	Flu B	**8578**	720	**3540**	1008	300	489	1761	**5555**	128
M	Flu B	**7500**	1292	**2882**	1798	559	1007	977	**4818**	353

2-Ads

Case ID	Infected by	H2.Jap.57 G	H5.VN.04 G	H5.Ind.05 E	H5.Ind.05 G	H7.NED.03 G	H7.SH.13 G	H7.NY.16 E	H9.HK.09 G	H13.DE.04 G
E	A(H1N1)pdm09	**4179**	367	1544	391	65	146	380	**3335**	127
F	A(H3N2)	511	100	686	159	64	131	321	95	86
G	A(H3N2)	114	67	174	74	95	163	986	150	87
H	A(H3N2)	**8848**	150	1380	196	147	338	398	**2619**	133
I	A(H3N2)	**4951**	339	1383	443	110	233	394	**3114**	120
J	A(H3N2)	**5180**	**2339**	1161	**2597**	79	147	516	**4691**	69
K	A(H3N2)	**3236**	592	1691	138	69	132	834	**114**	**2178**
L	Flu B	**8132**	546	1910	742	71	168	522	**5529**	117
M	Flu B	**6782**	393	496	390	57	131	308	**4839**	330

8-Ads

Case ID	Infected by	H2.Jap.57 G	H5.VN.04 G	H5.Ind.05 E	H5.Ind.05 G	H7.NED.03 G	H7.SH.13 G	H7.NY.16 E	H9.HK.09 G	H13.DE.04 G
E	A(H1N1)pdm09	**2509**	278	355	260	53	101	120	1744	108
F	A(H3N2)	ND	ND	ND	ND	ND	ND	ND	ND	ND
G	A(H3N2)	ND	ND	ND	ND	ND	ND	ND	ND	ND
H	A(H3N2)	**8090**	101	173	145	109	356	114	**2401**	96
I	A(H3N2)	**3817**	237	436	304	84	199	161	**2303**	75
J	A(H3N2)	**3096**	134	117	160	68	174	162	**4629**	152
K	A(H3N2)	**2542**	72	183	77	53	100	162	95	65
L	Flu B	**3628**	61	129	153	47	83	107	396	73
M	Flu B	**5088**	66	128	69	44	113	77	**4060**	47

Color scale
50
100
200
400
600
800
1000
1500
2000
3000
4000
5000
6000
7000
8000
9000
10000

MFIs against H2, H5, H7, H9, and H13 HA higher than 2000 were highlighted in bold.
MFIs against H9.09 G in four out of nine S2 sera might be induced by exposures to H2 HA.
